# Phosphoglucomutase 1 inhibits hepatocellular carcinoma progression by regulating glucose trafficking

**DOI:** 10.1371/journal.pbio.2006483

**Published:** 2018-10-18

**Authors:** Guang-Zhi Jin, Yajuan Zhang, Wen-Ming Cong, Xueyuan Wu, Xiongjun Wang, Siyang Wu, Siyao Wang, Weiping Zhou, Shengxian Yuan, Hong Gao, Guanzhen Yu, Weiwei Yang

**Affiliations:** 1 Department of Pathology, Eastern Hepatobiliary Surgery Hospital, Second Military Medical University, Shanghai, China; 2 State Key Laboratory of Cell Biology, CAS Center for Excellence in Molecular Cell Science, Innovation Center for Cell Signaling Network, Shanghai Institute of Biochemistry and Cell Biology, Chinese Academy of Sciences, University of Chinese Academy of Sciences, Shanghai, China; 3 Shanghai Key Laboratory of Molecular Andrology, Shanghai Institute of Biochemistry and Cell Biology, Chinese Academy of Sciences, University of Chinese Academy of Sciences, Shanghai, China; 4 Department of Radiation Oncology, First Affiliated Hospital of Wenzhou Medical College, Wenzhou, Zhejiang, China; 5 The Third Department of Hepatic Surgery, Eastern Hepatobiliary Surgery Hospital, Second Military Medical University, Shanghai, China; 6 Department of Oncology, Longhua Hospital Affiliated to Shanghai University of Traditional Chinese Medicine, Shanghai, China; University of California at Los Angeles, United States of America

## Abstract

Glycogen metabolism commonly altered in cancer is just beginning to be understood. Phosphoglucomutase 1 (PGM1), the first enzyme in glycogenesis that catalyzes the reversible conversion between glucose 1-phosphate (G-1-P) and glucose 6-phosphate (G-6-P), participates in both the breakdown and synthesis of glycogen. Here, we show that PGM1 is down-regulated in hepatocellular carcinoma (HCC), which is associated with the malignancy and poor prognosis of HCC. Decreased PGM1 expression obstructed glycogenesis pathway, which leads to the increased flow of glucose into glycolysis, thereby promoting tumor cell proliferation and HCC development. The loss of forkhead box protein J2 (FOXJ2), at least partly due to low genomic copy number in HCC, releases cellular nucleic acid-binding protein (CNBP), a nucleic acid chaperon, to bind to and promote G-quadruplex formation in PGM1 promoter and therefore decreases PGM1 expression. In addition, integrated analyses of PGM1 and FOXJ2 expression provide a better prediction for the malignance and prognosis of HCC. This study establishes a tumor-suppressive role of PGM1 by regulating glucose trafficking and uncovers a novel regulatory mechanism of PGM1 expression.

## Author summary

Hepatocellular carcinoma (HCC) is the most common type of primary liver cancer in adults. Sorafenib is the only clinically approved systemic drug for patients with advanced HCC. Identification of novel targets and biomarkers will provide new therapeutic strategies for advanced HCC and better prognostic prediction. Phosphoglucomutase (PGM) is an evolutionarily conserved enzyme that regulates one of the most important pathways in glucose metabolis—catalyzing the bidirectional interconversion of glucose 1-phosphate (G-1-P) and glucose 6-phosphate (G-6-P). In this study, we identify PGM1 as a metabolic tumor suppressor. Its expression allocates more glucose to glycogenesis, which reduces the glycolytic intermediates for biosynthesis, thereby impairing HCC progression. We delineate the mechanism of PGM1 down-regulation in HCC, finding that forkhead box protein J2 (FOXJ2) loss releases cellular nucleic acid-binding protein (CNBP) to bind to and modify the DNA structure of PGM1 promoter, thereby inhibiting PGM1 expression. Immunohistochemical analyses of human HCC tumors indicate that low FOXJ2 and PGM1 expression correlates with the malignancy and poor progression of human HCC. These results also suggest that the activation of residual PGM1 may impair HCC development through switching glycolysis to glycogenesis.

## Introduction

Hepatocellular carcinoma (HCC) is the most common type of primary liver cancer in adults and is the sixth most prevalent cancer and the third most frequent cause of cancer-related death [1]. Due to the rapid progression of HCC, most HCC patients are diagnosed at advanced stage, the 5-year survival rate of advanced HCC patients is as low as 25% to 39%, and the recurrence rate is approximately 80% [2]. Unfortunately, sorafenib is the only clinically approved systemic drug for patients with advanced HCC [3], and the Food and Drug Administration (FDA) recently expanded label indications for regorafenib to include treating patients with advanced HCC whose disease has progressed on the standard of care, sorafenib [4]. Therefore, identification of novel targets and predictors through studying the molecular basis underlying the pathogenesis of HCC will provide new therapeutic strategies for advanced-stage HCC and provide better methods for outcome prediction.

Altered metabolism has been commonly observed in various types of human cancers, especially in HCC. Tissue metabolomics and targeted phospholipid analyses show that HCC is characterized by increased glycolysis, attenuated mitochondrial oxidation, increased arachidonic acid synthesis, and increased choline catabolism and phospholipid metabolism [5,6]. Sorafenib-resistant HCC cells show higher glutamine metabolism and reductive glutamine carboxylation, which was accompanied by increased glucose-derived pentose phosphate pathway and glutamine-derived lipid biosynthetic pathways [7]. In addition, the interaction between KIAA1199 and glycogen phosphorylase kinase β-subunit (PHKB) or glycogen phosphorylase brain form (PYGB) can regulate glycogen breakdown to promote the survival of HCC cells under serum-free conditions [8]. These results strongly suggest the important role of deregulated metabolism in HCC development. However, how these metabolic pathways are altered and how these alterations contribute to the malignancy of HCC remain largely unknown.

Phosphoglucomutase (PGM) is an evolutionarily conserved enzyme that regulates one of the most important glucose trafficking points, catalyzing the bidirectional interconversion of glucose 1-phosphate (G-1-P) and glucose 6-phosphate (G-6-P). In one direction, G-1-P produced from glycogen catabolism is converted to G-6-P, the first intermediate in glycolysis. In the other direction, conversion of G-6-P to G-1-P generates a substrate for synthesis of uracil-diphosphate glucose (UDP-glucose), which is required for synthesis of a variety of cellular constituents, such as glycoproteins [9]. There are four PGM isozymes in human, including PGM1, PGM2, PGM3, and PGM5, which are encoded by different genes. In most cell types, PGM1 is predominant, representing about 90% of total PGM activity. It has been shown that PGM1 is glycosylated in response to heat shock and carbon source in *Saccharomyces cerevisiae* [10]. PGM1 is also phosphorylated by p21-activated kinase 1 (Pak1) at threonine 446, which significantly increases PGM1 enzymatic activity [11]. Under repetitive glucose depletion, depletion of PGM1 decreases glycogen content and the rates of glycogenolysis and glycogenesis, subsequently suppressing the proliferation of breast and cervical cancer cells [12]. However, the regulatory mechanism underlying PGM1 expression and how PGM1 expression contributes to HCC progression remain unclear.

In this study, we investigate the role and regulation of PGM1 expression in HCC. Our findings uncover a new mechanism that forkhead box protein J2 (FOXJ2)–up-regulated PGM1 expression inhibits tumor cell glycolysis, thereby impairing HCC growth.

## Results

### PGM1 is down-regulated in HCC and inversely correlates with HCC malignance

**Fig 1:**
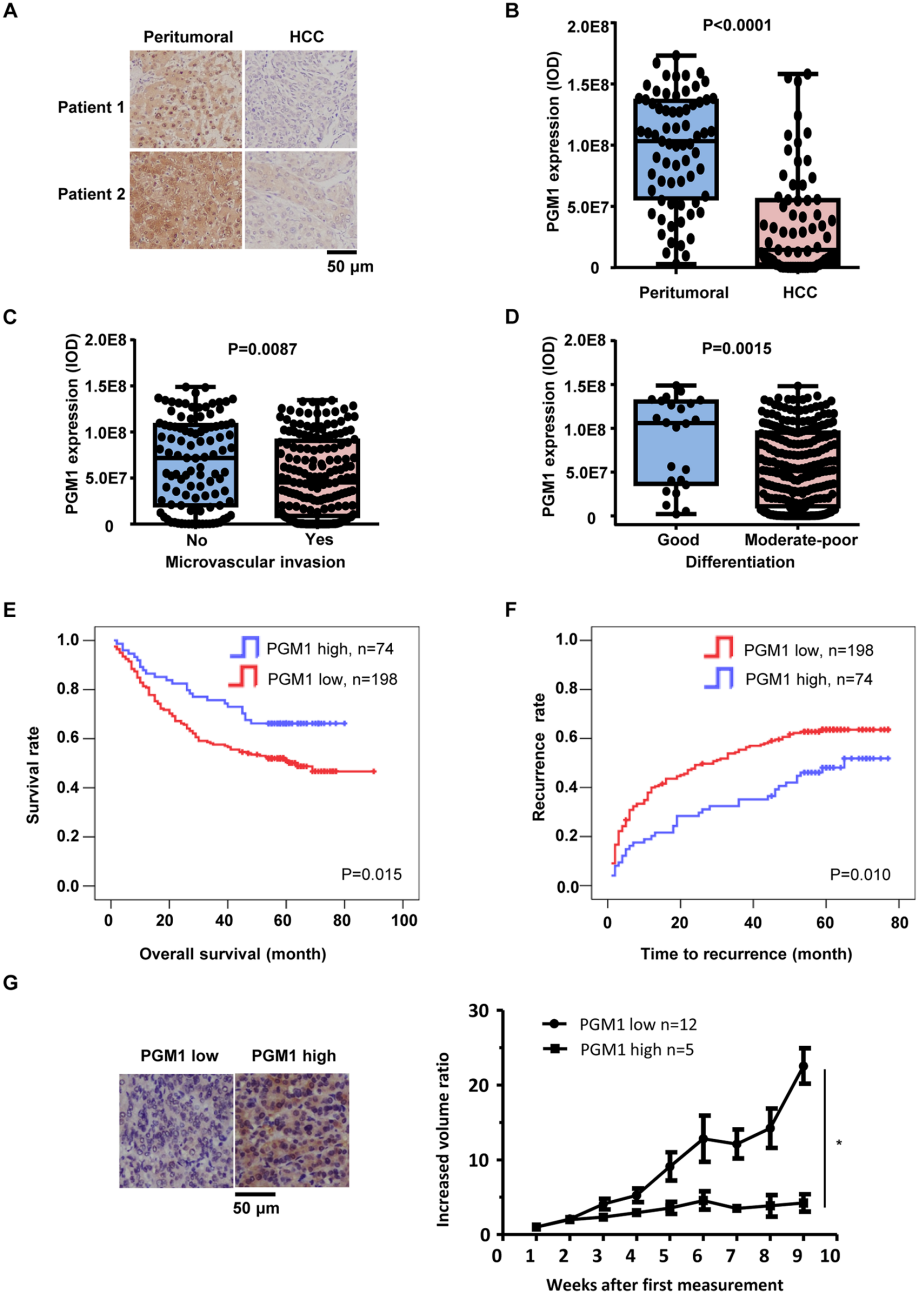
PGM1 is down-regulated in HCC and inversely correlates with HCC malignance. (A–B) IHC staining with anti-PGM1 antibody was performed on 69 pairs of HCC tumor tissues and corresponding peritumoral tissues. Representative images of tumors from two HCC patients are shown (panel A). Semiquantitative scoring was performed (panel B; paired *t* test, two-tailed, *P* < 0.0001). (C) IHC staining with anti-PGM1 antibody was performed on 272 tumor tissues from HCC patients with microvascular invasion (*n* = 169) or without microvascular invasion (*n* = 103). Semiquantitative scoring was performed (Mann-Whitney test, *P* = 0.0087). (D) IHC staining with anti-PGM1 antibody was performed on 272 HCC tumors with moderate to poor differentiation (*n* = 248) or with good differentiation (*n* = 24). Semiquantitative scoring was performed (Mann-Whitney test, *P* = 0.0018). (E–F) Kaplan-Meier curves of overall survival (panel E) and cumulative recurrence rates (panel F) were performed on 272 HCC patients after surgery. The optimal cutoff value of PGM1 staining is 9.6 × 10^7^, as determined by X-title software. Significance was determined by the log-rank test for both overall survival and cumulative recurrence rates. (G) PGM1 expression in 17 HCC PDX tumors was measured. Representative images of PGM1 expression from two PDX tumors (left panel) and tumor growth curves (right panel) are shown. The data of tumor growth were adapted from one of our previous studies. Underlying data can be found in S1 Data. HCC, hepatocellular carcinoma; IHC, immunohistochemistry; IOD, integrated optical density; PDX, patient-derived xenograft; PGM1, phosphoglucomutase 1.

To investigate the role of PGM1 in HCC, we performed immunohistochemistry (IHC) experiments to examine PGM1 expression in 69 pairs of tumor tissues and corresponding peritumoral tissues from HCC patients (Fig 1A). The specificity of anti-PGM1 antibody was validated by a protein-blocking assay with recombinant PGM1 (S1A Fig). Semiquantitative analyses of IHC staining showed that PGM1 expression was much lower in tumor tissues than that in paired peritumoral tissues (Fig 1B). Moreover, IHC analyses of PGM1 expression in 272 HCC patients showed that PGM1 expression was much lower in the tumors from HCC patients with microvascular invasion (*n* = 169) than that in the tumors from HCC patients without microvascular invasion (*n* = 103) (Fig 1C). Similarly, PGM1 expression was much lower in the tumors with moderate to poor differentiation (*n* = 248) than that in the tumors with good differentiation (*n* = 24) (Fig 1D). In addition, we compared overall survival duration or time to recurrence of 272 HCC patients with low staining versus those with high staining of PGM1 expression. The data showed that patients with low PGM1 expression in tumors had much lower median survival and much shorter time to recurrence than those with high PGM1 expression (Fig 1E and 1F). Collectively, these results strongly suggest the important role of PGM1 in the clinical behavior of HCC.

To further define the relationship between PGM1 expression and HCC aggressiveness, we examined PGM1 expression in 17 HCC patient-derived xenograft (PDX) tumors using IHC staining and compared the PGM1 levels with the progression of these PDX tumors (the data of tumor growth were adapted from one of our previous studies [13]). The results showed that the PDX tumors with low PGM1 expression (*n* = 12) had more rapid progression than the PDX tumors with high PGM1 expression (*n* = 5) (Fig 1G).

Because PGM1 is a metabolic enzyme, we next examined the enzymatic activity and the expression of PGM1 in 5 primary HCC tumors and a panel of HCC cell lines. As shown in S1B and S1C Fig, the enzymatic activity of PGM1 correlates with its protein levels in those primary tumors and cell lines.

In addition, we also tested whether PGM1 expression correlated with some commonly amplified or mutated oncogenes and tumor suppressors in HCC. Transcription analyses of HCC RNA-sequencing data (The Cancer Genome Atlas, PanCancer Atlas) by cBioPortal (http://www.cbioportal.org) showed that PGM1 mRNA expression had no or low correlation with the mRNA expressions of *CTNNB1* (r = 0.255), *MYC* (r = 0.009), *IGF2* (r = −0.276), *TGFA* (r = −0.288), *TP53* (r = −0.374), or *RB1* (r = −0.11) genes (S1D Fig). Besides, given that *CTNNB1* and *TP53* are frequently mutated in HCC, we analyzed *PGM1* mRNA expression between wild-type (WT) or mutant (MT) groups, which showed that *PGM1* mRNA expression was slightly higher in MT *CTNNB1* samples compared to WT *CTNNB1*, and *PGM1* mRNA expression showed no difference between WT *TP53* and MT *TP53* samples (S1E and S1F Fig). These results suggest that PGM1 does not have a simple mRNA regulation relationship with those altered oncogenes or tumor suppressors.

### PGM1 inhibits tumor cell proliferation and HCC development

**Fig 2:**
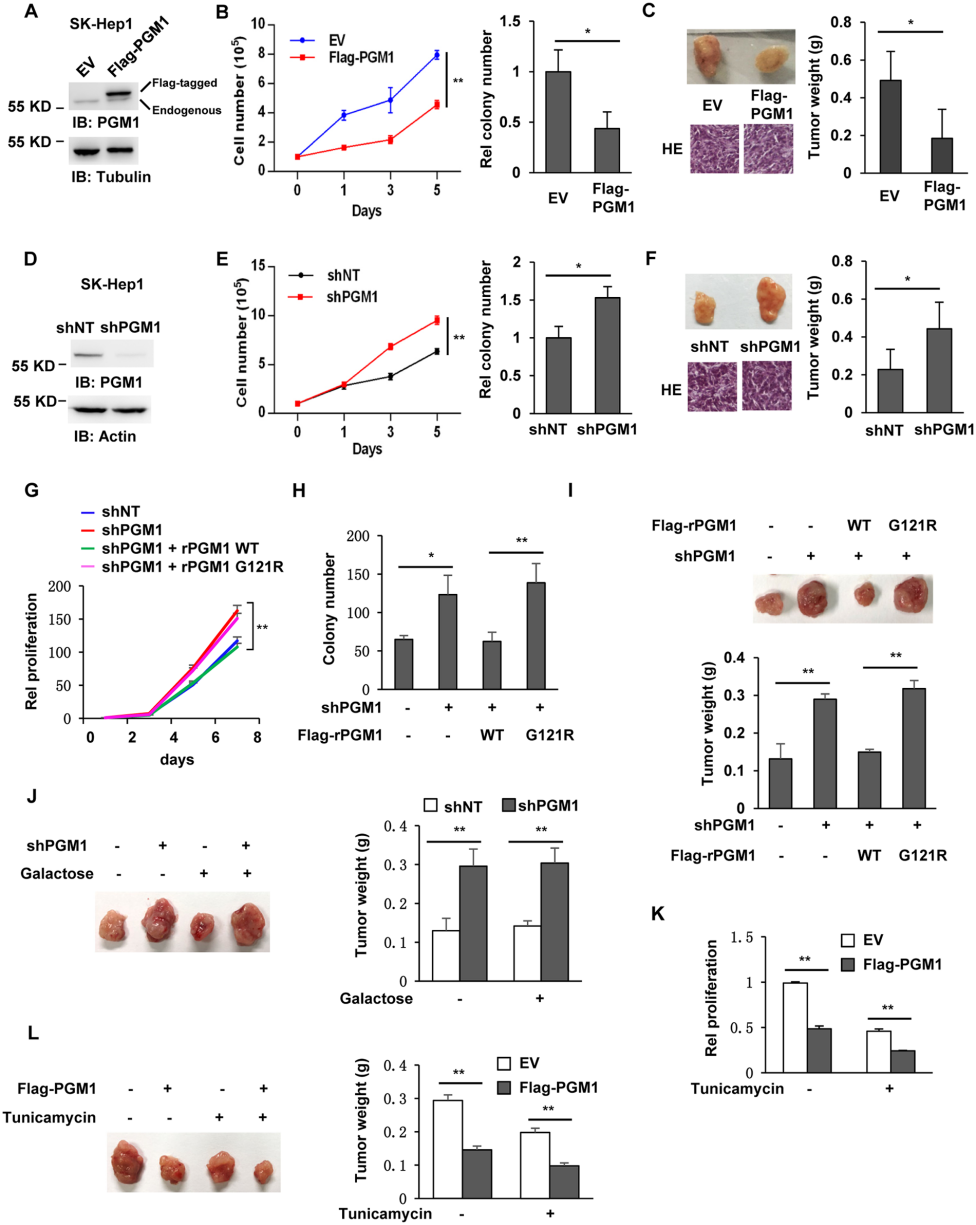
PGM1 inhibits tumor cell proliferation and tumor growth. Immunoblotting analyses were performed with the indicated antibodies. (A–C) SK-Hep1 cells were infected with the lentivirus expressing EV or Flag-PGM1. Immunoblotting analyses were performed in these cells (panel A). Proliferation (left panel) and colony formation (right panel) were examined in these cells (panel B). These cells were subcutaneously injected into randomized athymic nude mice (5 mice per group). Nine weeks after the injection, tumors were dissected for HE staining or weight measurement. Representative images of HE staining of dissected tumors are shown in left panel. Quantitative analyses of dissected tumor weights were performed (right panel). Data represent the means ± SD of 5 mice (panel C). (D–F) SK-Hep1 cells were infected with the lentivirus expressing shNT or shPGM1. IB analyses were performed in these cells (panel D). Proliferation (left panel) and colony formation (right panel) were examined in these cells (panel E). These cells were subcutaneously injected into randomized athymic nude mice (5 mice per group). At 30 days after the injection, tumors were dissected for HE staining or weight measurement. Representative images of HE staining of dissected tumors are shown in left panel. Quantitative analyses of dissected tumor weights were performed (right panel). Data represent the means ± SD of 5 mice (panel F). (G–I) SK-Hep1 cells were depleted of endogenous PGM1 and rescued with Flag-rPGM1 WT or G121R. Proliferation of these cells was determined by SRB assay (panel G). Colony formation was examined in these cells (panel H). These cells were subcutaneously injected into randomized athymic nude mice (5 mice per group). At 21 days after the injection, tumors were dissected for weight measurement (panel I). Representative images of dissected tumors are shown in upper panel. Quantitative analyses of dissected tumor weights are shown in lower panel. Data represent the means ± SD of 5 mice. (J) SK-Hep1 cells stably expressing shNT or shPGM1 were subcutaneously injected into randomized athymic nude mice (5 mice per group), and D-galactose was administered via oral gavage at 0.1 g/kg body weight per day for 3 weeks. Then, tumors were dissected for weight measurement. Representative images of dissected tumors are shown in left panel. Quantitative analyses of dissected tumor weights were performed (right panel). Data represent the means ± SD of 5 mice. (K–L) SK-Hep1 cells stably expressing EV or Flag-PGM1 were treated with or without 0.1 ug/ml Tunicamycin, and cell proliferation was measured (panel K). These cells were subcutaneously injected into randomized athymic nude mice (5 mice per group), and 0.5 mg/kg body weight Tunicamycin was administered by intraperitoneal injection twice a week. At 30 days after the inoculation, tumors were dissected for weight measurement. Representative images of dissected tumors are shown in left panel. Quantitative analyses of dissected tumor weights were performed (right panel). Data represent the means ± SD of 5 mice. Underlying data can be found in S1 Data. EV, empty vector; HE, hematoxylin–eosin; IB, immunoblotting; PGM1, phosphoglucomutase 1; shNT, nontargeting shRNA; shPGM1, shRNA against PGM1; shRNA, short hairpin RNA; SRB, sulforhodamine B; WT, wild-type.

To determine the role of PGM1 in HCC, we generated SK-Hep1 or Huh7 HCC cell lines stably overexpressing empty vector (EV) or Flag-PGM1 (Fig 2A and S2A Fig). As shown in Fig 2B and S2B Fig, PGM1 overexpression greatly inhibited the proliferation and colony formation of tumor cells. We further subcutaneously injected these genetically modified SK-Hep1 cells into randomized athymic nude mice. Nine weeks after implantation, we measured the weights of the dissected tumors, which showed that the mice injected with the cells expressing EV had rapid tumor growth. In contrast, PGM1 overexpression significantly impaired the tumor growth of SK-Hep1 cells (Fig 2C).

We next depleted endogenous PGM1 in SK-Hep1 or Huh7 cells using short hairpin RNA (shRNA) (Fig 2D and S2C Fig). Growth curve and colony formation (Fig 2E and S2D Fig) assays showed that PGM1 depletion enhanced cell proliferation and colony formation of HCC cells. In addition, we subcutaneously injected SK-Hep1 cells with or without PGM1 depletion into randomized athymic nude mice. At 30 days after implantation, we measured the weights of dissected tumors and found that PGM1 depletion accelerated tumor growth of HCC cells (Fig 2F).

As shown in S1C Fig, HepG2 cells had much higher PGM1 expression than SK-Hep1 cells. So, we depleted PGM1 in HepG2 cells (S2E Fig) and examined the proliferation and tumor growth in these cells, which showed that depletion of PGM1 also greatly promoted the proliferation and tumor growth of HepG2 cells (S2F and S2G Fig). Taken together, these results reveal a new role of PGM1 as tumor suppressor to inhibit HCC progression.

To exclude off-target effect of shPGM1, we depleted PGM1 in SK-Hep1 cells using another PGM1 shRNA (shPGM1-2). Depletion efficiency of shPGM1-2 is a bit lower than that of shPGM1 (S2H Fig). We next examined the proliferation of these two PGM1-depleted cells, which showed that both shPGM1 and shPGM1-2 enhanced tumor cell proliferation in a depletion efficiency-dependent manner (S2I Fig).

To test whether the enzymatic activity of PGM1 is required for antitumoral functions of PGM1, we constructed the enzyme-dead MT PGM1 G121R, which had much lower activity than PGM1 WT (S2J Fig). We next depleted endogenous PGM1 in SK-Hep1 cells and rescued these cells with shRNA-resistant (r) PGM1 WT or G121R (S2K Fig). Growth curves and colony formation assays showed that rescued expression of rPGM1 WT, but not rPGM1 G121R, abrogated PGM1 depletion-increased tumor cell proliferation (Fig 2G), colony formation (Fig 2H), and tumor growth (Fig 2I). These results indicate that the enzymatic activity of PGM1 is essential for PGM1-inhibited HCC development.

HCC with microvascular invasion has lower expression of PGM1 (Fig 1C). Thus, we wondered whether loss of PGM1 promoted the invasive ability of tumor cells. To test this hypothesis, we examined the migration and invasion of SK-Hep1 cells with PGM1 overexpression or depletion. As shown in S2L and S2M, neither PGM1 overexpression nor PGM1 depletion could affect tumor cell migration and invasion.

PGM1 deficiency is an inherited metabolic disorder in humans (CDG syndrome type 1t [CDG1T]), which is also a congenital disorder of protein N-glycosylation. Affected patients show multiple disease phenotypes, including dilated cardiomyopathy, exercise intolerance, and hepatopathy. Previous case reports in PGM1-CDG patients receiving oral D-galactose showed clinical improvement [14]. Thus, we next tested whether oral galactose supplementation could reverse low PGM1-promoted HCC progression. SK-Hep1 cells with or without PGM1 depletion were subcutaneously injected into randomized athymic nude mice, followed by the oral administration of galactose. The weights of dissected tumors showed that galactose supplementation had no effects on tumor growth of HCC cells expressing nontargeting shRNA (shNT) or shRNA against PGM1 (shPGM1). Moreover, PGM1-depletion–promoted tumor growth could not be abrogated by such supplementation either (Fig 2J). These results suggest that PGM1-deficiency–promoted tumor growth is glycosylation independent.

To further verify whether glycosylation is involved in PGM1-regulated HCC progression, we treated SK-Hep1 cells stably overexpressing PGM1 with or without Tunicamycin, a specific inhibitor of N-linked glycosylation. The efficacy of Tunicamycin was validated by the increased GRP78 expression (S2N Fig), as reported previously [15]. As shown in Fig 2K and 2L, Tunicamycin treatment had no effects on PGM1-overexpression–inhibited tumor cell proliferation and HCC progression.

### PGM1 deficiency switches glycogenesis to glycolysis to promote tumor cell proliferation

**Fig 3:**
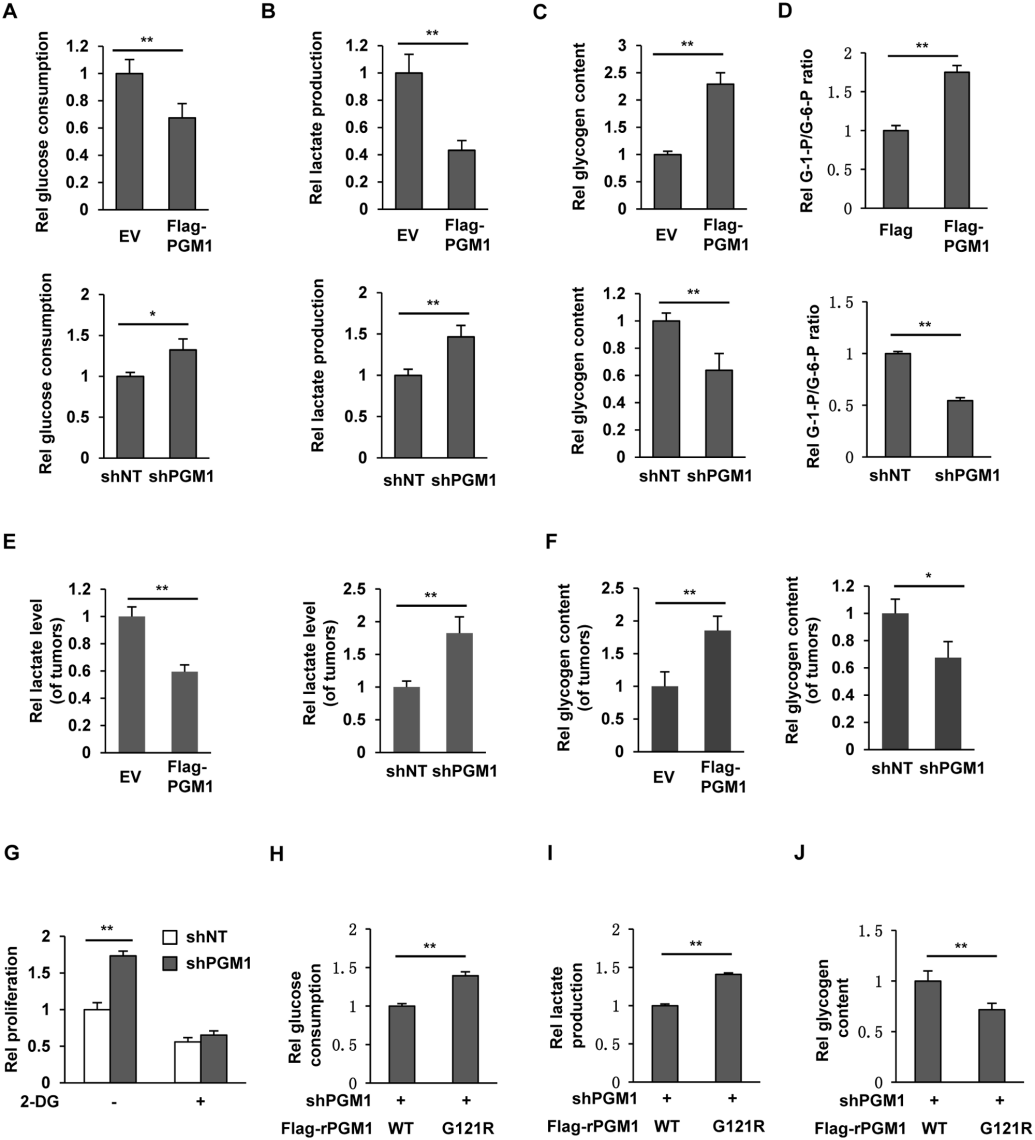
PGM1 enhances glycogen synthesis but inhibits aerobic glycolysis. (A–D) SK-Hep1 cells were infected with the lentivirus expressing EV, Flag-PGM1, shNT, or shPGM1. The culture media of these cells were collected for analysis of glucose consumption (panel A) and lactate production (panel B). Glycogen content (panel C) and G-1-P/G-6-P ratio (panel D) of these cells were measured. Data represent the means ± SD of 3 independent experiments. (E–F) SK-Hep1 cells stably expressing EV, Flag-PGM1, shNT, or shPGM1 were subcutaneously injected into randomized athymic nude mice (5 mice per group). Thirty days later, dissected tumors were homogenized and deproteinized for the measurement of lactate content using L-Lactate Assay kit (panel E). Glycogen content was measured in dissected tumors (panel F). Data represent the means ± SD of 5 mice. (G) SK-Hep1 cells stably expressing shNT or shPGM1 were treated with or without 0.5 mM 2-DG, and cell proliferation was measured. Data represent the means ± SD of 3 independent experiments. (H–J) The culture media of endogenous PGM1-depleted SK-Hep1 cells rescued with Flag-rPGM1 WT or G121R were collected for analysis of glucose consumption (panel H) and lactate production (panel I). Glycogen content (panel J) of these cells was measured. Data represent the means ± SD of 3 independent experiments. Underlying data can be found in S1 Data. 2-DG, 2-Deoxyglucose; EV, empty vector; G-1-P, glucose 1-phosphate; G-6-P, glucose 6-phosphate; PGM1, phosphoglucomutase 1; shNT, nontargeting shRNA; shPGM1, shRNA against PGM1; shRNA, short hairpin RNA; WT, wild-type.

PGM1 catalyzes the reversible transfer of phosphate between the 1 and 6 positions of glucose, thereby regulating the important glucose trafficking between glycogen synthesis and glycolysis. We therefore wondered whether PGM1 inhibits tumor growth through regulating this glucose trafficking. Glucose, lactate, and glycogen assays showed that overexpression of PGM1 greatly inhibited glucose consumption and lactate production but increased glycogen content of SK-Hep1 cells, while depletion of PGM1 significantly enhanced glucose consumption and lactate production but decreased glycogen content of tumor cells (Fig 3A–3C). Similar results were obtained in HepG2 cells, showing that depletion of PGM1 greatly enhanced glucose consumption and lactate production but decreased glycogen content of tumor cells (S3A–S3C Fig). Such PGM1-regulated glucose trafficking was also evidenced by the ratio of G-1-P/G-6-P in SK-Hep1 cells. As shown in Fig 3D, PGM1 overexpression elevated the ratio of G-1-P/G-6-P in tumor cells, while this ratio was reduced by PGM1 depletion.

In addition, we also examined the levels of lactate and glycogen in the tumors dissected from the mice implanted with SK-Hep1 cells stably overexpressing EV or PGM1, or in the tumors dissected from the mice implanted with SK-Hep1 cells stably expressing shNT or shPGM1. The experiments showed that PGM1 overexpression greatly decreased the levels of lactate but increased the levels of glycogen in the tumors. In contrast, PGM1 depletion significantly increased the levels of lactate but decreased the levels of glycogen in the tumors (Fig 3E and 3F). Taken together, these results demonstrate that PGM1 expression switches glycolysis to glycogenesis.

To further define the role of PGM1 in such switch from glycolysis to glycogenesis, we compared the activities of glycolysis and glycogenesis between SK-Hep1 cells (low PGM1) and HepG2 cells (high PGM1). The experiments showed that HepG2 cells had less glucose consumption and lactate production but higher glycogen content than SK-Hep1 cells (S3D–S3F Fig). Moreover, HepG2 cells had higher G-1-P levels and G-1-P/G-6-P ratio but lower G-6-P levels than SK-Hep1 cells (S3G–S3I Fig), and more N-linked glycans were detected in HepG2 cells than in SK-Hep1 cells (S3J Fig). Consistently, HepG2 had much slower proliferation rate and tumor growth than SK-Hep1 cells (S3K and S3L Fig).

To investigate whether PGM1-deficiency–promoted tumor cell proliferation requires glycolysis, we treated SK-Hep1 cells stably expressing shNT or shPGM1 with or without the glycolysis inhibitor 2-Deoxyglucose (2-DG). The cell proliferation assays showed that 2-DG treatment almost completely abrogated PGM1-depletion–enhanced tumor cell proliferation (Fig 3G). Furthermore, SK-Hep1 cells (low PGM1) are more sensitive to the treatment of 2-DG than HepG2 cells (high PGM1) (S3M Fig). These results strongly suggest that glycolysis is essential for PGM1-deficiency–promoted tumor cell proliferation.

In addition, we also examined whether the enzymatic activity of PGM1 is required for such glucose trafficking regulation. Glucose, lactate, and glycogen assays showed that SK-Hep1 cells rescued with rPGM1 G121R had much higher glucose consumption and lactate production but much lower glycogen content than those cells rescued with rPGM1 WT (Fig 3H–3J), underscoring the importance of PGM1 activity in regulating such glucose metabolism.

### FOXJ2 enhances PGM1 promoter activity to increase PGM1 expression

**Fig 4:**
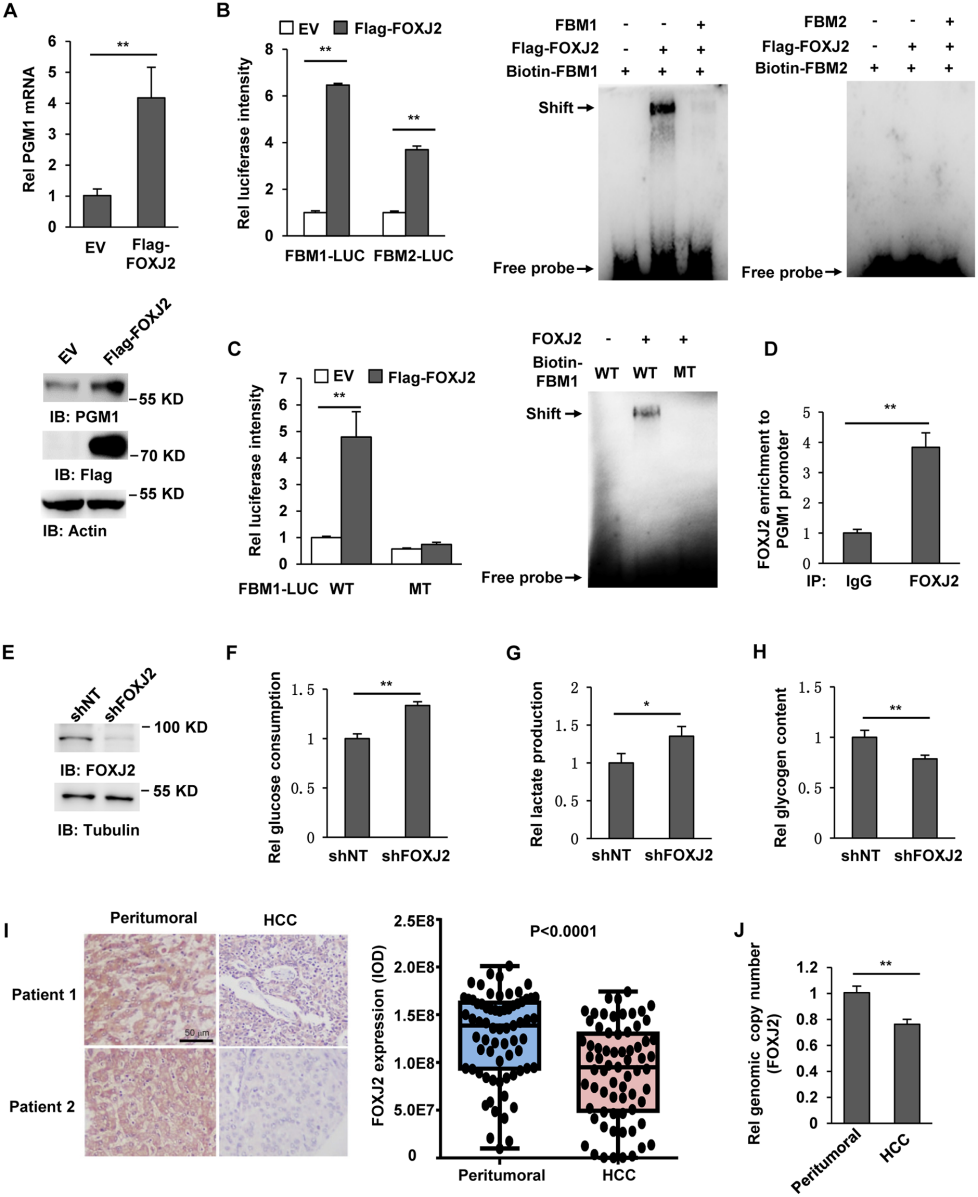
FOXJ2 enhances pgm1 promoter activity to increase PGM1 expression. Immunoblotting analyses were performed with the indicated antibodies. (A) SK-Hep1 cells were stably transfected with EV or Flag-FOXJ2. Real-time PCR (upper panel) and immunoblotting analyses (lower panel) were performed to examine mRNA and protein levels of PGM1. Data represent the means ± SD of 3 independent experiments. (B) The luciferase reporter construct containing either FBM1 or FBM2 fragment was cotransfected with EV or Flag-FOXJ2 into SK-Hep1 cells. The relative levels of luciferase activity of Firefly were normalized to the levels of luciferase activity of control Renilla. Data represent the mean ± SD of 3 independent experiments (left panel). Flag-FOXJ2 protein was immunoprecipitated from SK-Hep1 cells stably expressing Flag-FOXJ2 using anti-Flag antibody and eluted with Flag peptides. EMSA was performed by mixing eluted Flag-FOXJ2 and biotin-labeled FBM1 or FBM2 (Biotin-FBM1 or Biotin-FBM2) oligonucleotides (middle and right panel). (C) The luciferase reporter construct containing either FBM1 WT or FBM1 MT fragment was cotransfected with EV or Flag-FOXJ2 into SK-Hep1 cells. The relative levels of luciferase activity of Firefly were normalized to the levels of luciferase activity of control Renilla. Data represent the mean ± SD of 3 independent experiments (left panel). Flag-FOXJ2 protein was immunoprecipitated from SK-Hep1 cells stably expressing Flag-FOXJ2 using anti-Flag antibody and eluted with Flag peptides. EMSA was performed by mixing eluted Flag-FOXJ2 and Biotin-FBM1 WT or Biotin-FBM1 MT oligonucleotides (right panel). (D) ChIP analyses were performed with an anti-FOXJ2 antibody in SK-Hep1 cells. Real-time PCR analysis was carried out to amplify PGM1 promoter. Data represent the means ± SD of 3 independent experiments. (E–H) SK-Hep1 cells were infected with the lentivirus expressing shNT or shFOXJ2 (panel E). The culture media of these cells were collected for analysis of glucose consumption (panel F) and lactate production (panel G). Glycogen content (panel H) of these cells was measured. Data represent the means ± SD of 3 independent experiments. (I) IHC staining with anti-FOXJ2 antibody was performed in 69 pairs of tumor tissues and corresponding peritumoral tissues from HCC patients. Representative images of tumor tissues and paired peritumoral tissues are shown in left panel. Semiquantitative scoring was performed (right panel, paired *t* test, two-tailed, *P* < 0.0001). (J) Real-time PCR was performed to examine relative genomic copy number of FOXJ2 in paired tumor tissues and corresponding peritumoral tissues from 9 HCC patients. Data represent the means ± SD of 9 patients. Underlying data can be found in S1 Data. ChIP, chromatin immunoprecipitation; EMSA, electrophoretic mobility shift assay; EV, empty vector; FBM1, FOXJ2-binding motif 1; FOXJ2, forkhead box protein J2; HCC, hepatocellular carcinoma; IHC, immunohistochemistry; MT, mutant; PGM1, phosphoglucomutase 1; shNT, nontargeting shRNA; shPGM1, shRNA against PGM1; shRNA, short hairpin RNA; WT, wild-type.

To explore how PGM1 expression is regulated, we analyzed PGM1 promoter sequence using the Transcription Factor Search Portal (http://gpminer.mbc.nctu.edu.tw/index.php). Among the predicted transcription factors, FOXJ2 was the first transcriptional factor that might regulate PGM1 expression and was also reported to suppress tumor growth. FOXJ2 is a member of the forkhead transcription factor family [16] and was down-regulated in HCC and extrahepatic cholangiocarcinoma [17,18]. We therefore tested whether FOXJ2 regulates PGM1 expression. We generated SK-Hep1 cells stably expressing EV or Flag-FOXJ2. Real-time PCR and immunoblotting analyses showed that FOXJ2 overexpression increased mRNA and protein levels of PGM1 (Fig 4A). We analyzed PGM1 promoter sequence and found two FOXJ2-binding motifs (FBMs), including FBM1 or FBM2 (S4A Fig). To test whether FOXJ2 regulates PGM1 promoter activity, we constructed a luciferase reporter system containing repeated FBM1 or FBM2 (FBM1-Luc or FBM2-Luc), respectively. As shown in Fig 4B, FOXJ2 overexpression enhanced luciferase activities of both FBM1-Luc (over 6-fold) and FBM2-Luc (over 3-fold). But electrophoretic mobility shift assay (EMSA) using biotin-labelled FBM1 or FBM2 (biotin-FBM1 or biotin-FBM2) oligonucleotides showed that FOXJ2 only bound to FBM1 but not FBM2 (Fig 4B). We speculated that the activation of FBM2-Luc by FOXJ2 may be indirect. FOX proteins are a big family of transcription factors. The activation of FBM2-Luc without directly binding to FBM2 might be due to that FOXJ2 activates another FOX protein that directly binds to FBM2, thereby activating FBM2-Luc. However, this hypothesis needs further experiments to clarify. We next mutated 5′-TAAACA-3′ in FBM1 (FBM1 WT), which exactly matched the Fox family conserved binding motif (T/CAAACA), into 5′-TGCGCG-3′ (FBM1 MT) and constructed luciferase reporter system using these repeated sequences of FBM1 WT or MT (FBM1 WT-Luc or FBM1 MT-Luc). The intensities of luciferases showed that FOXJ2 overexpression greatly increased the luciferase activity of FBM1 WT-Luc, while it failed to increase that of FBM1 MT-Luc. The EMSA assays consistently showed that FOXJ2 could bind to FBM1 WT, but not FBM1 MT (Fig 4C). The association of FOXJ2 with PGM1 promoter was also supported by the chromatin immunoprecipitation (ChIP) assays using anti-FOXJ2 antibody in SK-Hep1 cells, showing that FOXJ2 was recruited to the PGM1 promoter (Fig 4D). To confirm the specificity of FOXJ2-regulated PGM1 promoter, we further tested whether a well-known tumor suppressor, p53—which was also predicted to regulate PGM1 promoter—could bind to PGM1 promoter. The ChIP assays using anti-p53 antibody showed that p53, not like FOXJ2, was not recruited to the promoter of PGM1 (S4B Fig).

We next tested whether FOXJ2 also regulates the glucose trafficking. Endogenous FOXJ2 was depleted in SK-Hep1 cells (Fig 4E). Glucose, lactate, and glycogen assays showed that FOXJ2 depletion enhanced glucose consumption and lactate production but reduced glycogen content of tumor cells (Fig 4F–4H). Taken together, these results demonstrate that FOXJ2 up-regulates PGM1 expression to switch glycolysis to glycogenesis by activating PGM1 promoter.

To further define the relationship between FOXJ2 expression and HCC malignancy, we examined FOXJ2 expression in 69 pairs of tumor tissues and peritumoral tissues from HCC patients. Semiquantitative analyses of IHC staining of these tissues showed that FOXJ2 expression was dramatically decreased in HCC tumor tissues compared to paired peritumoral tissues (Fig 4I). We next examined genomic copy number of FOXJ2 in tumor tissues and corresponding peritumoral tissues from 9 HCC patients, which showed that HCC tumor tissues had much lower genomic copy number of FOXJ2 than paired peritumoral tissues (Fig 4J), suggesting that decreased FOXJ2 expression is at least partially due to such low genomic copy number. It was reported that FOXJ2 expression was down-regulated by transforming growth factor (TGF-β1) in non–small-cell lung cancer [19]. We next tested whether TGF-β1 regulates FOXJ2 expression in HCC. As shown in S4C Fig, TGF-β1 treatment did not affect FOXJ2 expression in SK-Hep1 and HepG2 cells, suggesting that various mechanisms of FOXJ2 down-regulation were involved in different types of cancers.

### FOXJ2 disrupts the binding of cellular nucleic acid-binding protein to PGM1 promoter

**Fig 5:**
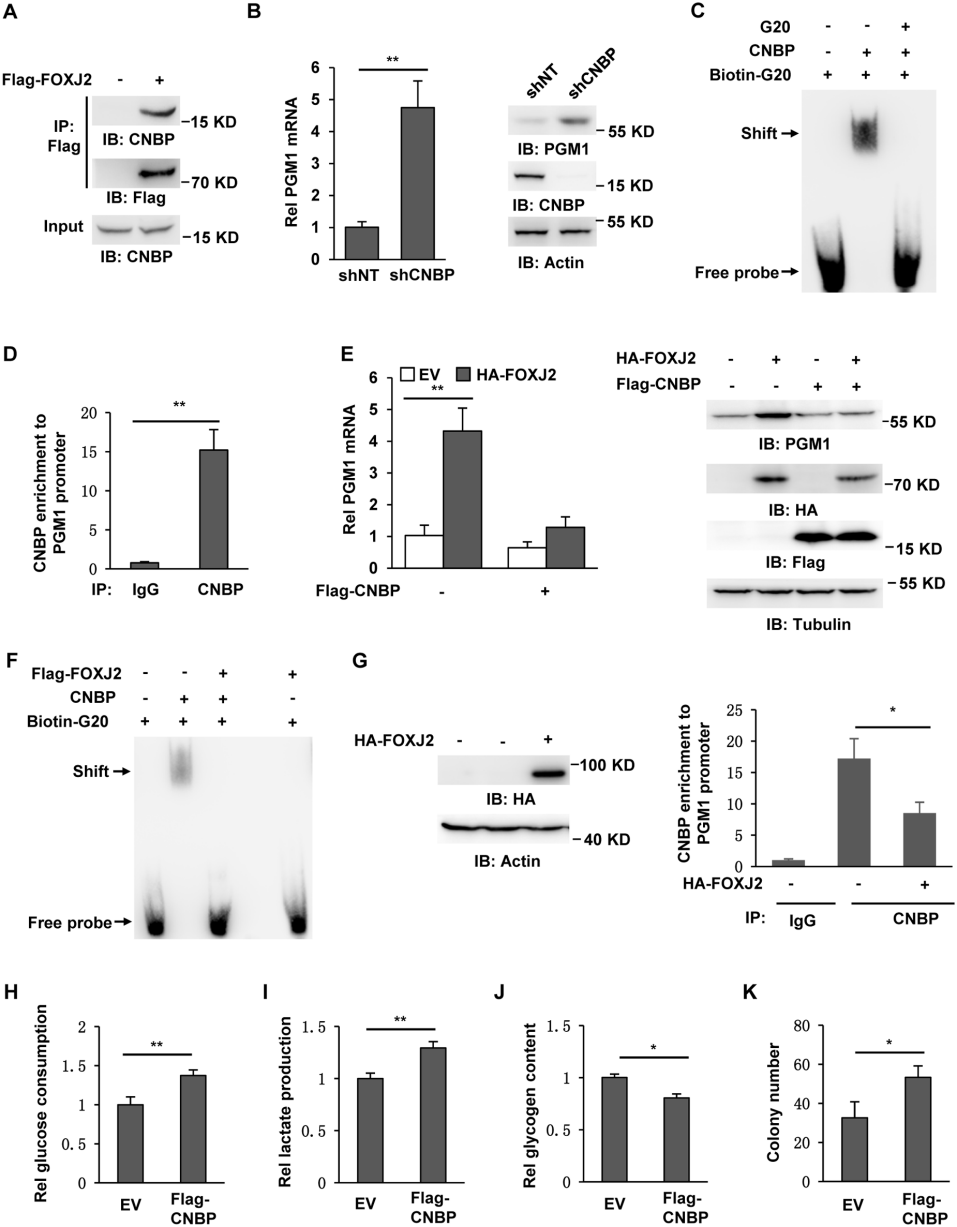
FOXJ2 disrupts the binding of CNBP to PGM1 promoter. Immunoblotting analyses and coimmunoprecipitation were performed with the indicated antibodies. (A) SK-Hep1 cells were transiently transfected with EV or Flag-FOXJ2. (B) Real-time PCR (left panel) and immunoblotting analyses (right panel) were performed to examine mRNA and protein levels of PGM1 in SK-Hep1 cells stably expressing shNT or shCNBP. Data represent the means ± SD of 3 independent experiments. (C) EMSA was carried out by mixing bacteria-purified recombinant human CNBP with biotin-G20 oligonucleotides. (D) ChIP analyses with an anti-CNBP antibody were performed in SK-Hep1 cells. Data represent the means ± SD of 3 independent experiments. (E) SK-Hep1 cells stably expressing EV or HA-FOXJ2 were infected with a lentivirus expressing EV or Flag-CNBP. Real-time PCR (left panel) and immunoblotting analyses (right panel) were performed to examine mRNA and protein levels of PGM1. Data represent the means ± SD of 3 independent experiments. (F) Flag-FOXJ2 was immunoprecipitated from SK-Hep1 cells stably expressing Flag-FOXJ2 and eluted with Flag peptides. EMSA was performed by mixing bacteria-purified recombinant CNBP and biotin-G20 oligonucleotides in the presence or absence of eluted Flag-FOXJ2 protein. (G) SK-Hep1 cells were infected with a lentivirus expressing EV or HA-FOXJ2. ChIP analyses with an anti-CNBP antibody were performed in these cells. Data represent the means ± SD of 3 independent experiments. (H–K) SK-Hep1 cells were infected with the lentivirus expressing EV or Flag-CNBP. The culture media of these cells were collected for analysis of glucose consumption (panel H) and lactate production (panel I). Glycogen content (panel J) and colony formation (panel K) of these cells were measured. Data represent the means ± SD of 3 independent experiments. Underlying data can be found in S1 Data. ChIP, chromatin immunoprecipitation; CNBP, cellular nucleic acid-binding protein; EMSA, electrophoretic mobility shift assay; EV, empty vector; FOXJ2, forkhead box protein 2; HA-FOXJ2, HA-tagged FOXJ2; IP, immunoprecipitation; PGM1, phosphoglucomutase 1; shNT, non-targeting shRNA; shPGM1, shRNA against PGM1; shRNA, short hairpin RNA.

It has been shown that the transcriptional outputs of FOX proteins are fine-tuned through interaction with other pioneer factors and insulator proteins [20]. To identify these pioneer factors or insulator proteins, we performed a mass spectrum analysis of FOXJ2-associated proteins in SK-Hep1 cells stably expressing Flag-FOXJ2. In the descending order of FOXJ2-associated proteins, cellular nucleic acid-binding protein (CNBP) was the first protein that directly binds to DNA and regulates gene transcription (S5A Fig). We further validated the interaction of these two proteins using coimmunoprecipitation assay with anti-Flag antibody in SK-Hep1 cells stably expressing Flag-FOXJ2. The experiments showed that FOXJ2 interacted with CNBP (Fig 5A).

G-rich sequences that contain stretches of tandem guanines can form four-stranded, intramolecular stable DNA structures called G-quadruplexes (termed G4s). CNBP is a nucleic acid chaperone that binds the G4-forming G-rich sequence located within the promoters of targeted genes [21]. CNBP has been initially described as a DNA-binding protein acting as a negative transcriptional regulator in the coordinated control of cholesterol metabolism [22]. So, we depleted endogenous CNBP in SK-Hep1 cells using shRNA and examined PGM1 expression. Real-time PCR and immunoblotting analyses showed that CNBP knockdown increased PGM1 transcription and protein expression (Fig 5B). We further analyzed PGM1 promoter sequence and found a G-rich (G20) sequence (5′-GGGCTGGCGCGGAGGGAGGG-3′), which might be a potential binding motif of CNBP. To test this hypothesis, we performed EMSA by mixing purified recombinant CNBP and biotin-labelled G20 oligonucleotides. The experiments showed that CNBP directly bound to this biotin-G20 motif and unlabeled G20 oligonucleotides competitively inhibited CNBP binding to biotin-G20 motif (Fig 5C). We further performed ChIP assays in SK-Hep1 cells using anti-CNBP antibody and found that CNBP was recruited to G20 region of PGM1 promoter (Fig 5D).

We next tested whether FOXJ2 increases PGM1 expression through CNBP. SK-Hep1 cells stably expressing EV or HA-FOXJ2 were infected with the lentivirus expressing EV or Flag-CNBP. As shown in Fig 5E, CNBP overexpression greatly abrogated FOXJ2-up-regulated PGM1 mRNA and protein expression. Moreover, we performed EMSA by mixing recombinant CNBP and biotin-G20 in the presence or absence of Flag-FOXJ2 precipitated from SK-Hep1 cells stably expressing Flag-FOXJ2. The experiments showed that FOXJ2 disrupted the binding of CNBP to G20 without directly interacting with G20 (Fig 5F). This result was also supported by the ChIP assay using anti-CNBP antibody in SK-Hep1 cells stably expressing EV or HA-FOXJ2, which showed that FOXJ2 overexpression dramatically attenuated the recruitment of CNBP to PGM1 promoter (Fig 5G). Collectively, these results demonstrate that FOXJ2 interacts with CNBP and prevents its association with G20 motif in PGM1 promoter, thereby promoting PGM1 expression.

We further examined the effects of CNBP overexpression on glycolysis, glycogenesis, and proliferation of SK-Hep1 cells (S5B Fig). As shown in Fig 5H–5J, CNBP overexpression enhanced glucose consumption and lactate production but decreased glycogen content of tumor cells. Consequently, the proliferation of tumor cells was greatly accelerated by CNBP overexpression, as determined by colony formation assay (Fig 5K).

In addition, we compared CNBP expression between SK-Hep1 (low PGM1) and HepG2 (high PGM1) cells and found that these two HCC cells had comparable levels of CNBP expression (S5C Fig). Moreover, IHC analyses using anti-CNBP antibody also showed that primary HCC tumors had slightly lower CNBP expression than paired peritumoral tissues (S5D Fig).

### FOXJ2-dependent PGM1 up-regulation inhibits glycolysis, cell proliferation, and HCC development

**Fig 6:**
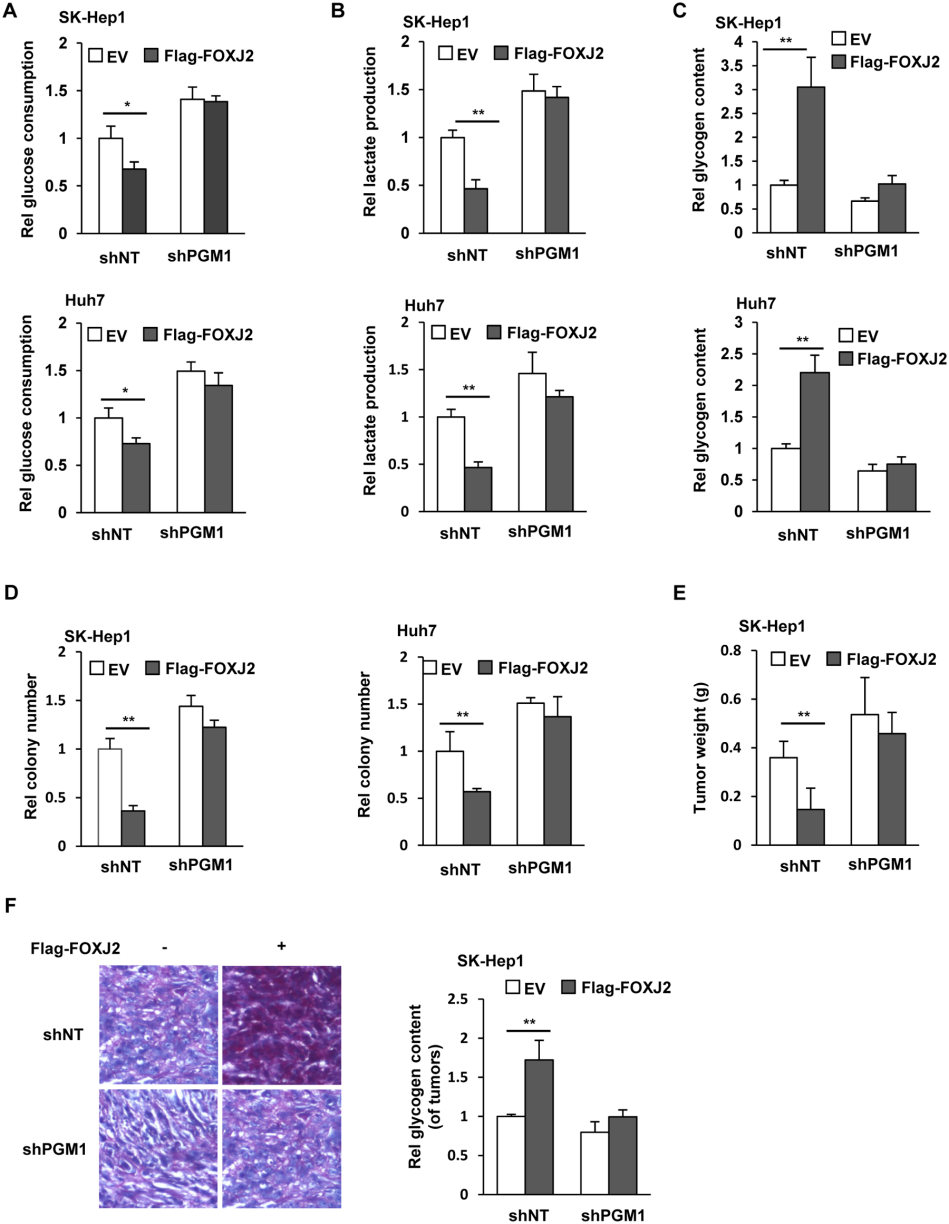
FOXJ2-up-regulated PGM1 expression inhibits glycolysis, cell proliferation, and HCC development. (A–D) SK-Hep1 or Huh7 cells stably expressing shNT or shPGM1 were infected with a lentivirus expressing EV or Flag-FOXJ2. The culture media of these cells were collected for analysis of glucose consumption (panel A) and lactate production (panel B). Glycogen content (panel C) and colony formation (panel D) of these cells were measured. Data represent the means ± SD of 3 independent experiments. (E–F) SK-Hep1 cells stably expressing shNT or shPGM1 were infected with a lentivirus expressing EV or Flag-FOXJ2. These cells were subcutaneously injected into randomized athymic nude mice (6 mice per group). At 30 days after the injection, tumors were dissected for weight (panel E) and glycogen content (panel F) measurement. Representative images of Periodic Acid-Schiff staining of the tumors were presented (panel F, left panel). Glycogen content of the tumors was measured. Data represent the means ± SD of 6 mice (panel F, right panel). Underlying data can be found in S1 Data. EV, empty vector; FOXJ2, forkhead box protein J2; HCC, hepatocellular carcinoma; PGM1, phosphoglucomutase 1; shNT, nontargeting shRNA; shPGM1, shRNA against PGM1; shRNA, short hairpin RNA.

To investigate the biological function of FOXJ2-dependent PGM1 expression, we overexpressed EV or Flag-FOXJ2 in SK-Hep1 or Huh7 cells with or without PGM1 depletion (S6A Fig). As shown in Fig 6A and 6B, FOXJ2 overexpression inhibited glucose consumption and lactate production in tumor cells, which was reversed by PGM1 depletion. In contrast, glycogen content was increased by FOXJ2 overexpression, which was also abrogated by PGM1 depletion (Fig 6C). Consistently, FOXJ2 overexpression inhibited the colonies formation, and PGM1 depletion almost completely abrogated such phenotypes of tumor cells (Fig 6D).

To further determine the role of FOXJ2-dependent PGM1 regulation in tumorigenesis of HCC cells, we subcutaneously injected SK-Hep1 cells stably expressing EV or Flag-FOXJ2 with or without PGM1 depletion into randomized athymic nude mice. The weights of dissected tumors showed that FOXJ2 overexpression greatly impaired tumor growth, while PGM1 depletion almost completely rescued the tumor growth inhibited by FOXJ2 overexpression (Fig 6E). Periodic Acid-Schiff staining and glycogen assays of dissected tumors showed that FOXJ2 overexpression increased glycogen storage in the tumors. PGM1 depletion abrogated such increase of glycogen content (Fig 6F).

In addition, we compared FOXJ2 expression between SK-Hep1 and HepG2 cells, which showed that HepG2 had much higher FOXJ2 protein levels than SK-Hep1 cells (S6B Fig). We further overexpressed PGM1 in HepG2 cells (S6C Fig) and found that the proliferation of HepG2 cells could also be inhibited by PGM1 overexpression (S6D Fig), which is similar to SK-Hep1 cells (Fig 2B), suggesting that PGM1 is downstream of FOXJ2 and required for FOXJ2-inhibited tumor cell proliferation.

Taken together, these results demonstrate that FOXJ2-dependent PGM1 up-regulation shunt glycolysis to glycogenesis, thereby decreasing the glycolytic intermediates for tumor cell proliferation and HCC development.

### PGM1 and FOXJ2 expression correlates with the prognosis of HCC patients

**Fig 7:**
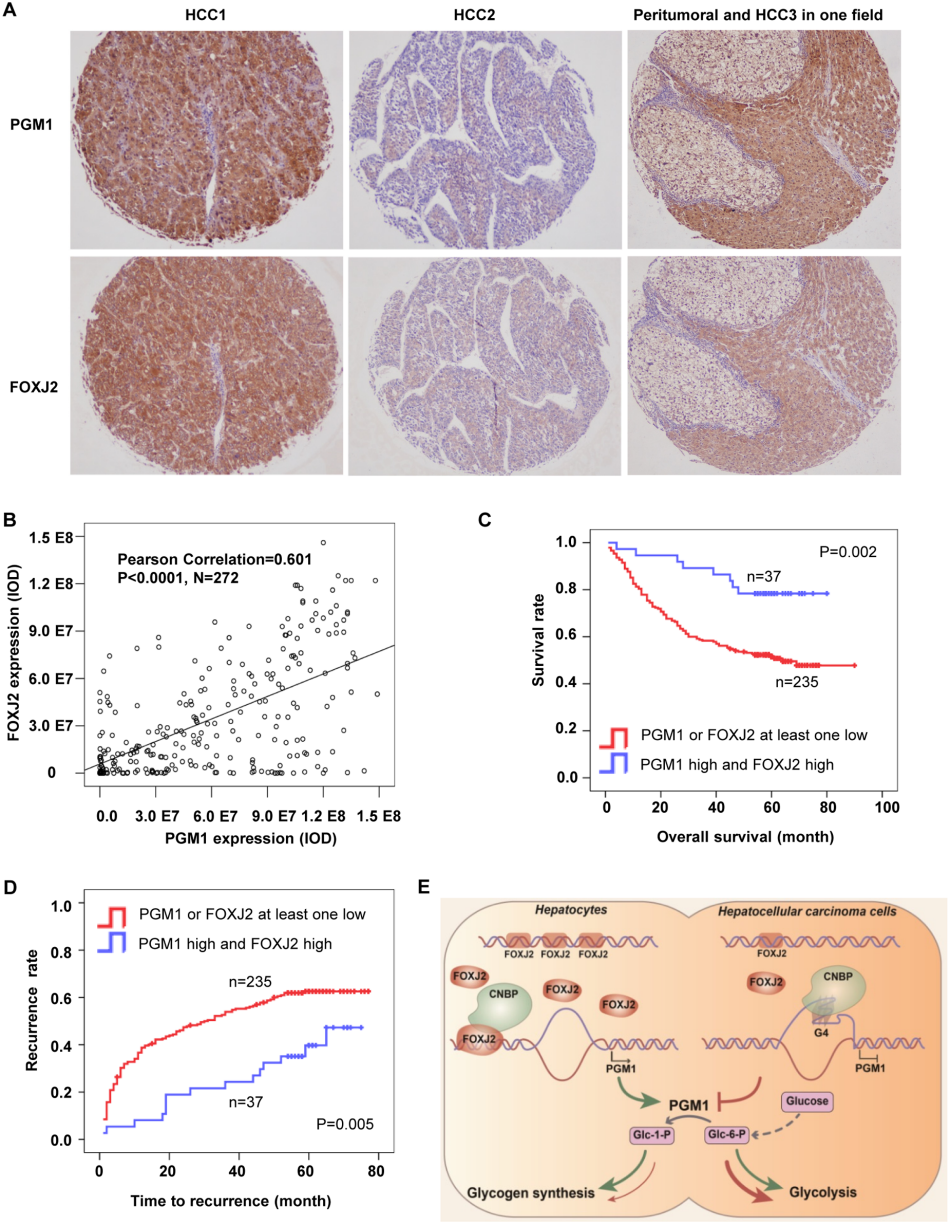
PGM1 and FOXJ2 expression correlates with the prognosis of HCC patients. IHC staining using anti-PGM1 or anti-FOXJ2 antibodies were performed in 272 HCC tissues, respectively. (A) Representative images of IHC staining of tumor tissues from three HCC patients were shown. (B) Pearson correlation analysis was performed to determine correlation between PGM1 and FOXJ2 expression in HCC tissues (Pearson correlation coefficient = 0.601, *P* < 0.0001). (C–D) Kaplan-Meier curves of overall survival (panel C) and cumulative recurrence rates (panel D) were performed on HCC patients. The optimal cutoff values of PGM1 staining (9.6 × 10^7^) and FOXJ2 staining (27.3 × 10^7^) were determined by X-title software. Significance was determined by the log-rank test for both overall survival and cumulative recurrence rates. (E) Schematic model of mechanism that FOXJ2-dependent PGM1 up-regulation inhibits HCC growth. Upon sufficient extracellular glucose, FOXJ2 binds to PGM1 promoter and interacts with CNBP, which subsequently prevents CNBP-mediated G4-DNA formation in PGM1 promoter, thereby increasing PGM1 expression. This FOXJ2-dependent PGM1 up-regulation enhances glycogen synthesis in HCC cells, which consequently reduces glucose flux into glycolysis for biosynthesis processes and therefore inhibits tumor cell proliferation. Underlying data can be found in S1 Data. CNBP, cellular nucleic acid-binding protein; FOXJ2, forkhead box protein J2; HCC, hepatocellular carcinoma; PGM1, phosphoglucomutase 1.

To define the clinical significance of FOXJ2-dependent PGM1 expression, we performed IHC analyses of PGM1 and FOXJ2 expression using anti-PGM1 and anti-FOXJ2 antibodies. The experiment showed that PGM1 protein levels correlated with FOXJ2 protein levels (Fig 7A). Semiquantification of the IHC staining showed that the correlation of these two proteins was significant (Pearson correlation = 0.601, *P* < 0.0001, *n* = 272) (Fig 7B). As described in Fig 1E and 1F, decreased PGM1 expression correlated with poor prognosis of HCC patients. FOXJ2 expression has also been suggested to correlate with poor prognosis of HCC patients [18]. To determine prognostic potential of integrated analyses of PGM1 and FOXJ2 expression, we divided 272 HCC patients into 2 groups. The patients in group I had low expression of at least one protein of PGM1 and FOXJ2; the patients in group II had high expression of both proteins. Kaplan–Meier analyses of overall survival clearly showed that the patients in group II had much better prognosis compared to the patients in group I (Fig 7C). Comparison of time to recurrence between these 2 groups also consistently showed that group II patients had much longer time to recurrence than group I patients (Fig 7D). Moreover, univariate and multivariate Cox regression analysis indicated that the two-protein panel of PGM1 and FOXJ2 combination was an independent prognostic factor for postoperative survival and recurrence in HCC patients (S7A Fig).

In addition, we analyzed the correlation between the IHC intensities of PGM1, FOXJ2, or CNBP and hepatitis B surface antigen (HBsAg) status (representing HBV infection). Chi-squared analysis showed that PGM1, FOXJ2, or CNBP expression did not correlate with HBsAg status (S7B Fig).

## Discussion

Deregulated glycogen metabolism commonly exists in human cancers. PGM1 is a key enzyme in glycogen metabolism by regulating glucose trafficking. However, how PGM1 expression is regulated and how this expression regulates HCC development remain unclear. In the present study, we demonstrate that decreased FOXJ2 expression releases CNBP to promote G4-DNA formation in PGM1 promoter, thereby decreasing PGM1 expression. Decreased PGM1 expression inhibits glycogen synthesis to spare more glucose for glycolysis and therefore supports rapid growth of HCC (Fig 7E). These findings reveal a unique role of PGM1 as a tumor suppressor in HCC and uncover a novel mechanism of FOXJ2/CNBP-regulated PGM1 expression as well as implicate prognostic potential of integrated analyses of both FOXJ2 and PGM1 expression.

Although the role of aerobic glycolysis in cancer has been extensively studied, abnormalities in other metabolic pathways are only just being understood in cancer. Glycogen metabolism is one such pathway. The presence of glycogen has been described in various cancer cells and tumors, where glycogen content was inversely correlated with proliferation rate [23,24]. However, the role and regulation of glycogen metabolism in cancer progression remain poorly understood. Inhibition of glycogen degradation has been linked to reduced activity of biosynthetic pathways and antioxidant defenses of the cell. For example, glycogen phosphorylase (PYGL) depletion is associated with a reduction in cell proliferation, decreased functioning of the pentose phosphate pathway, and increased levels of reactive oxygen species [25]. As for the regulation of glycogen metabolism, hypoxia has been shown to induce glycogen storage as well as PGM1 expression, which requires hypoxia-inducible factor 1 (HIF-1) and HIF-2. This hypoxia-induced glycogen stores are rapidly mobilized in noncancer or cancer cells that are starved of glucose. And glycogenolysis allows these cells to confront and survive glucose deprivation [26]. However, the authors did not answer whether HIF-1 and HIF-2 directly regulate PGM1 expression and whether PGM1 expression contributes to cancer progression. In our study, by contrast, we show that, with sufficient extracellular glucose, PGM1 expression enhances glycogen synthesis in tumors cells, which consequently reduces glucose flux into glycolysis for biosynthesis processes and therefore inhibits the proliferation of HCC cells. FOXJ2 interacts with CNBP on PGM1 promoter, which subsequently prevents CNBP-mediated G4-DNA formation of the sequences in the promoter, thereby increasing PGM1 expression.

PGM1 regulates an important glucose trafficking point by catalyzing interconversion of G-1-P and G-6-P. PGM1 deficiency is an inherited metabolic disorder in humans (CDG1T). Affected patients show multiple disease phenotypes, including dilated cardiomyopathy, exercise intolerance, and hepatopathy, reflecting the central role of the enzyme in glucose metabolism. In contrast, little is known about the role of PGM1 in cancer. In the present study, we demonstrate that PGM1 expression in HCC cells inhibits cell proliferation and tumor growth by diverting glucose into glycogen synthesis from glycolysis with sufficient extracellular glucose. On the contrary, PGM1 depletion in HCC cells promotes tumor cell proliferation and tumor growth by inhibiting glycogen synthesis to spare more glucose for glycolysis. These findings reveal a unique function of PGM1 as a tumor suppressor to inhibit the proliferation of HCC cells upon sufficient glucose supply. A recent study shows that depletion of PGM1 decreases glycogen content and the rates of glycogenolysis and glycogenesis, which subsequently suppresses the proliferation of breast and cervical cancer cells under long-term repetitive glucose depletion [12]. This study suggests that PGM1 also plays a prosurvival role in tumor cells upon glucose deprivation. Collectively, these findings illustrate that PGM1 can play a protumoral or antitumoral role in a context-dependent manner.

PGM1 is regulated by a set of mechanism. For example, PGM1 is glycosylated in response to heat shock and carbon source in *S*. *cerevisiae*, which does not regulate its enzymatic activity but rather is involved in the location of the protein [10]. PGM1 is also phosphorylated by Pak1 at threonine 446, which increases PGM1 enzymatic activity [11]. In addition, hypoxia increases PGM1 expression to increase glycogen storage in HIF-1- and HIF-2-dependent manner, although whether HIF directly regulates PGM1 expression is unclear. In the present study, we demonstrate that FOXJ2 directly binds to PGM1 promoter and interacts with CNBP, which prevents CNBP-mediated G4-DNA formation in PGM1 promoter, thereby increasing PGM1 expression. In HCC, reduced genomic copy number leads to decreased FOXJ2 expression, which releases CNBP to promote G4-DNA formation in PGM1 promoter, thereby inhibiting PGM1 expression.

FOXJ2 is a member of the forkhead transcription factor family [16] and was down-regulated in HCC and extrahepatic cholangiocarcinoma [17,18]. FOXJ2 has been suggested to function as a tumor suppressor gene in breast cancer cells, glioma cells, cholangiocarcinoma cells, HCC cells, and non–small-cell lung cancer, and low expression levels of FOXJ2 indicates poor prognosis of cancer patients [17,19,27,28]. However, whether FOXJ2 can regulate tumor cell metabolism to inhibit cancer progression remains unknown. Here, we show that FOXJ2 regulates glucose trafficking between glycolysis and glycogen metabolism by increasing PGM1 expression to inhibit tumor cell glycolysis and proliferation. To the best of our knowledge, our study provides the first demonstration of FOXJ2-regulating cancer cell metabolism.

Although altered glycogen metabolism commonly exists in various human cancers, the prognostic value of PGM1 in cancer has been rarely addressed except one study, which shows that PGM1 mRNA levels inversely correlate with human HCC and cirrhosis [29]. In this study, we analyzed PGM1 and FOXJ2 protein expression in the tumors from 272 human HCC patients using IHC and found that low PGM1 expression reflects poor prognosis of HCC patients after standard treatment. Moreover, as shown in Fig 7C and 7D, integrated analyses of the expression of both PGM1 and FOXJ2 proteins may more precisely predict the prognosis and recurrence rate of HCC patients. These results strongly suggest that the expression of both PGM1 and FOXJ2 may be a promising prognostic predictor of HCC patients.

## Materials and methods

### Ethics statement

The use of human tumor specimens and the database was approved by the Institutional Review Board at Eastern Hepatobiliary Surgery Hospital (EHBH; approval number: EHBHKY2014-03-006). Informed consent was obtained from all patients.

All mouse experiments were conducted in accordance with standard operating procedures approved by the Institutional Animal Care and Use Committee of the Shanghai Institute for Biological Sciences, Chinese Academy of Sciences.

### Materials

Mouse monoclonal antibody against Flag and anti-Flag M2 affinity gel were purchased from Sigma (St. Louis, MO). Rabbit monoclonal antibody against HA Tag and phospho-Smad2 (Ser465/467) were obtained from Cell Signaling Technology (Danvers, MA). Mouse monoclonal antibody against PGM1 and rabbit polyclonal antibody against FOXJ2 were purchased from Abcam (Cambridge, MA). Flag peptide and mouse monoclonal antibody against Actin were obtained from Abclonal Technology (Wuhan, China). Rabbit polyclonal antibody against CNBP and rabbit polyclonal antibody against GRP78 were obtained from Proteintech Group (Wuhan, China). Mouse monoclonal antibody against p53 were obtained from Santa Cruz Biotechnology (Santa Cruz, CA). D-Galactose and Tunicamycin were obtained from MedChemExpress (Monmouth Junction, NJ). TGFβ recombinant protein was bought from Peprotech (New Jersey). Puromycin and hygromycin were bought from EMD Biosciences (SanDiego, CA). In vitro DNA transfection reagent was purchased from Signagen Laboratories (Rockville, MD). GelCode Blue Stain Reagent was obtained from Pierce (Rockford, IL).

### Cell culture

SK-Hep1, Huh7, HepG2, 7721, and Hep3B HCC cells were obtained from the Type Culture Collection of the Chinese Academy of Sciences. Cell lines used in the experiments have been authenticated by short tandem repeat profiling. Mycoplasma contamination was examined using Cycleave PCR Mycoplasma Detection Kit (Takara). These cells were maintained in Dulbecco’s Modified Eagle Medium (DMEM) supplemented with 10% fetal bovine serum (FBS) and antibiotics.

### DNA constructs

PCR-amplified human PGM1, FOXJ2, and CNBP were cloned into pCDH-Flag, pcDH-HA, pcDNA3-HA, and pET-28a vectors. Synthetic FBM1 WT, FBM1 MT, and FBM2 DNA oligonucleotides were cloned into pGL3-promoter vectors. Mutations were made using the QuickChange site-directed mutagenesis kit (Stratagene, La Jolla, CA).

The pGIPZ shNT was generated with the control oligonucleotide 5′-GCTTCTAACACCGGAGGTCTT-3′. pGIPZ PGM1 shRNA was generated with 5′-TCGGTAGAAGCTTATGCTACA-3′ oligonucleotide. pGIPZ PGM1 shRNA-2 was generated with 5′-CTGCATCATTAGAAAAATCAA-3′ oligonucleotide. pGIPZ FOXJ2 shRNA was generated with 5′- TCGAACAACTACTACATGTAT-3′ oligonucleotide. pGIPZ CNBP shRNA was generated with 5′-CTGCACCAAAGTGAAGTGCTA-3′ oligonucleotide.

### Immunoprecipitation and immunoblotting analysis

Extraction of proteins with a modified buffer from cultured cells was followed by immunoprecipitation and immunoblotting with corresponding antibodies, as described previously [30].

### Cell proliferation assay

A total of 1 × 10^5^ cells in DMEM with 10% fetal calf serum were seeded per well in 6-well plate. Cell proliferation rates were determined by cell counting every 2 days. Data represent the mean ± SD of 3 independent experiments.

For SRB assay, 1,000 cells per well were plated in 96-well plate and cultured for indicated days. Cells were fixed with trichloroacetic acid and stained with SRB solution, followed by rinsing the plates four times with 1% acetic acid and solubilizing the protein-bound dye with 10 mM Tris base solution. The absorbance was measure at 510 nm. Data represent the mean ± SD of 3 independent experiments.

### Colony formation assay

For colony formation assay, 5,000 cells per well were plated in 6-well plate in triplicates and cultured for 14 days before staining viable colonies with crystal violet. Data represent the mean ± SD of 3 independent experiments.

### Glucose, lactate, and glycogen assays

A total of 2 × 10^5^ cells were seeded in 12-well plate, and the media were changed after 6 hours. Twenty hours later, culture media were collected for measurement of glucose and lactate using Glucose (GO) Assay Kit (Sigma) and Lactate Assay Kit (Biovision). Glycogen content of these cells was measured according to manufacturer instructions of Glycogen Assay Kit (Biovision). Glucose consumption, lactate production, and glycogen content were normalized by cell numbers. Data represent the mean ± SD of 3 independent experiments.

To measure lactate and glycogen content in tumors, dissected tumors were washed with cold PBS and homogenized, followed by centrifugation at 10,000 g for 10 minutes at 4°C. Lactate and glycogen content were measured according to manufacturer instructions of Lactate Assay Kit (Abcam) and Glycogen Assay Kit (Biovision).

### PAS staining

Subcutaneous tumors were dissected and paraffin-embedded. Periodic Acid-Schiff staining was performed according to manufacturer instructions of Periodic Acid-Schiff Stain Kit (Abcam).

### Purification of recombinant proteins

HIS-CNBP was expressed and purified in bacteria as described previously [31]. Briefly, BL21/DE3 cells were transformed with pET28a-CNBP. The expression of CNBP was induced by 0.2 mM IPTG for 24 hours at 16 °C. Cell lysates were incubated with Ni-NTA resin (Genescript) for 4 hours at 4 °C. HIS-CNBP protein was eluted with 500 mM imidazole and dialyzed against PBS.

### ChIP assay

ChIP was performed with indicated antibodies using SimpleChIP Enzymatic Chromatin IP Kits (Cell Signaling Technology) according to manufacturer instructions. Primer sequences used for the amplification of human *PGM1* promoter associated with FOXJ2 were 5′- TTGCTAATACCAGCCAATT -3′ (forward) and 5′- CCTAAGGCTCTACAATCAATAA -3′ (reverse). Primer sequences used for the amplification of human *PGM1* promoter associated with CNBP were 5′-CTTGCCCTATGACCGGGTTC-3′ (forward) and 5′-AAGTTCTCCGCGTAGTTGGC-3′ (reverse). Primer sequences used for the amplification of human *PGM1* promoter associated with p53 were 5′-GGTTTTGGAGCCAGACCAC-3′ (forward) and 5′-ACTTGCTGAGTGTAGCGTTTC-3′ (reverse).

### Electrophoretic mobility shift assay

EMSA was performed using Chemiluminescent EMSA Kit (Thermo) according to manufacturer instructions. Briefly, biotin-labeled oligonucleotides were mixed with indicated proteins in EMSA binding buffer at room temperature for 20 minutes. Samples were subjected to 5% polyacrylamide gel electrophoresis and transferred to nylon membrane. Crosslinked nylon membrane was subjected to an appropriately equipped CCD camera.

### Luciferase reporter assay

SK-Hep1 cells were cotransfected with the luciferase reporter vector pGL3-promoter containing FBM1 WT, FBM1 MT, or FBM2 and control Renilla plasmid. At 24 hours after transfection, cells were harvested for the measurement of luciferase activity. The relative levels of luciferase activity were normalized to the levels of luciferase activity of control Renilla plasmid. Data represent the mean ± SD of 3 independent experiments.

### Invasion and migration assays

Invasion assays were performed in 8 μm-pore transwell inserts with coated Matrigel, as instructed by the manufacturer (BD Biosciences, San Jose, CA). A total of 1 × 10^5^ cells were seeded in the upper chamber with serum-free medium while the bottom chambers were filled with medium containing 10% FBS. After 12 hours, the invaded cancer cells were stained with crystal violet, followed by solubilisation of crystal violet with 100 μl 33% acetic acid. Invasion ability was determined by optical density (OD570) measurement. Migration assay were performed the same as invasion assay without coated Matrigel. Data represent the mean ± SD of 3 independent experiments.

### PGM1 enzymatic activity assay

Cells were collected for measurement of PGM1 activity using Phosphoglucomutase Colorimetric Assay Kit (Biovision). Data represent the mean ± SD of 3 independent experiments.

### G-1-P and G-6-P assays

Cells were collected for measurement of G-1-P and G-6-P content using Glucose 1-Phosphate Colorimetric Assay Kit (Biovision) and Glucose 6-Phosphate Colorimetric Assay Kit (Biovision). Data represent the mean ± SD of 3 independent experiments.

### N-linked glycan assay

Extraction of N-linked glycans with PNGase F from cultured cells was followed by MALDI-TOF Mass Spectrometry, as described previously [32].

### Subcutaneous injection

In brief, 6-week-old randomized female athymic nude mice were subcutaneously injected with 1 × 10^6^ SK-Hep1 or HepG2 cells. Five mice were included in each experiment. Thirty days after injection, mice were euthanized, and tumors were dissected for the measure of tumor weight, tumor lactate, and glycogen content. All mice were housed in a specific pathogen-free environment at the Shanghai Institute of Biochemistry and Cell Biology.

### Patients and follow-up

Formalin-fixed paraffin-embedded (FFPE) specimens were obtained from EHBH, Second Military Medical University, Shanghai, China, and 341 patients were used in the present study. Tissue microarrays were constructed as reported previously [33]. We constructed 2 sets of tissue microarrays. One included HCC and paired peritumoral tissues from 69 patients. Another one included HCC tissues only from 272 patients who had completed follow-up. Patients underwent curative resection between 2003 and 2006 at EHBH, and patients were followed until October 2010.

### Tissue microarrays and IHC

Tissue microarrays and IHC and integrated optical density (IOD) measurement were performed as described previously [34].

### Statistical analysis

Statistical analyses were carried out with SPSS 13.0 software and GraphPad Prism 5.01. The significance of differential expression of indicated proteins between peritumoral tissues and tumoral tissues from 69 HCC patients was analyzed by the paired *t* test as described previously [35], and Mann–Whitney test was used for analyzing between nonpaired groups [34]. *P* < 0.05 was considered to be significant. *P* < 0.001 was considered to be extremely significant. For calculating the best cut-off points for over survival and time to recurrence, the X-Tile statistical package (version 3.5.0; Yale University, New Haven, CT) was used as described previously [36].

## Supporting information

S1 Fig PGM1 is down-regulated in HCC and inversely correlates with HCC malignance. Related to Fig 1. Immunoblotting analyses were performed with the indicated antibodies. (A) IHC analyses of HCC tumor tissues using anti-PGM1 antibody were performed with or without PGM1 blocking protein. (B) PGM1 expression (upper panel) and enzymatic activity (lower panel) were measured in 5 primary HCC tumors. Data represent the means ± SD of 3 independent experiments. (C) PGM1 expression (upper panel) and enzymatic activity (lower panel) were measured in a panel of HCC cell lines. Data represent the means ± SD of 3 independent experiments. (D) Transcriptional correlation analyses (Pearson’s correlation) of *PGM1* and some commonly amplified or mutated oncogenes or tumor suppressors in HCC. (E) *PGM1* mRNA expression was compared between WT *CTNNB1* and MT *CTNNB1* samples (Nonsense mutations were excluded). Mann-Whitney test, *P* = 0.0002. (F) *PGM1* mRNA expression was compared between WT *TP53* and MT *TP53* samples (Nonsense mutations were excluded). Mann-Whitney test, *P* = 0.1827. Underlying data can be found in S1 Data. CTNNB1, catenin beta-1; HCC, hepatocellular carcinoma; IHC, immunoblotting analyses; MT, mutant; PGM1, phosphoglucomutase 1; TP53, Cellular tumor antigen p53; WT, wild-type.
(TIF)

S2 Fig PGM1 inhibits tumor cell proliferation and tumor growth. Related to Fig 2. Immunoblotting analyses were performed with the indicated antibodies. (A–B) Huh7 cells were infected with the lentivirus expressing EV or Flag-PGM1. Immunoblotting analyses were performed in these cells (panel A). Proliferation (left panel) and colony formation (right panel) were examined in these cells (panel B). Data represent the means ± SD of 3 independent experiments. (C–D) Huh7 cells were infected with the lentivirus expressing shNT or shPGM1. Immunoblotting analyses were performed in these cells (panel C). Proliferation (left panel) and colony formation (right panel) were examined in these cells (panel D). Data represent the means ± SD of 3 independent experiments. (E–F) HepG2 cells were infected with the lentivirus expressing shNT or shPGM1. Immunoblotting analyses were performed in these cells (panel E). Proliferation (panel F) was examined in these cells using SRB assay. Data represent the means ± SD of 3 independent experiments. (G) Cells in panel E were subcutaneously injected into randomized athymic nude mice (five mice per group). At 30 days after the injection, tumors were dissected for weight measurement. Representative images of dissected tumors are shown in left panel. Quantitative analyses of dissected tumor weights are shown in right panel. Data represent the means ± SD of five mice. (H–I) SK-Hep1 cells were infected with the lentivirus expressing shNT, shPGM1-2 or shPGM1. Immunoblotting analyses (panel H) and proliferation (panel I) were performed in these cells. Data represent the means ± SD of 3 independent experiments. (J) SK-Hep1 cells were infected with the lentivirus expressing Flag-PGM1 WT or G121R. Flag-PGM1 proteins were immunoprecipitated using Flag beads and eluted with Flag peptides to determine PGM1 enzymatic activity. (K) SK-Hep1 cells were depleted of endogenous PGM1 and rescued with Flag-rPGM1 WT or G121R. Immunoblotting analyses were performed in these cells. (L–M) Migration (panel L) and invasion (panel M) of SK-Hep1 cells stably expressing EV, Flag-PGM1, shNT or shPGM1 were examined. (N) SK-Hep1 cells were treated with or without 0.1 ug/ml Tunicamycin for 24 hours, and immunoblotting analyses were performed in these cells. Underlying data can be found in S1 Data. EV, empty vector; PGM1, phosphoglucomutase 1; shNT, nontargeting shRNA; shPGM1, shRNA against PGM1; SRB, sulforhodamine; WT, wild-type.
(TIF)

S3 Fig PGM1 enhances glycogen synthesis but inhibits aerobic glycolysis. Related to Fig 3. Data represent the means ± SD of 3 independent experiments. (A–C) The culture media of HepG2 cells stably expressing shNT or shPGM1 were collected for analysis of glucose consumption (panel A) and lactate production (panel B). Glycogen content (panel C) of these cells were measured. (D–I) The culture media of SK-Hep1 and HepG2 cells were collected for analysis of glucose consumption (panel D) and lactate production (panel E). Glycogen content (panel F), G-1-P level (panel G), and G-6-P (panel H) of SK-Hep1 and HepG2 cells were measured. G-1-P/G-6-P ratio was calculated (panel I). (J) N-linked glycans of SK-Hep1 and HepG2 cells were measured. (K) Proliferation was examined in SK-Hep1 and HepG2 cells. (L) SK-Hep1 or HepG2 cells were subcutaneously injected into randomized athymic nude mice (five mice per group). At 35 days after the injection, tumors were dissected for weight measurement. Representative images of dissected tumors are shown in left panel. Quantitative analyses of dissected tumor weights are shown in right panel. Data represent the means ± SD of five mice. (M) SK-Hep1 or HepG2 cells were treated with or without 0.5 mM 2-DG, and proliferation of these cells was examined. Underlying data can be found in S1 Data. 2-DG, 2-Deoxyglucose; G-1-P, glucose 1-phosphate; G-6-P, glucose 6-phosphate; PGM1, phosphoglucomutase 1; shNT, nontargeting shRNA; shPGM1, shRNA against PGM1; shRNA, short hairpin RNA.
(TIF)

S4 Fig FOXJ2 enhances PGM1 promoter activity to increase PGM1 expression. Related to Fig 4. Immunoblotting analyses were performed with the indicated antibodies. (A) Sequences of two FOXJ2 binding motifs (FBM) 1 and 2 in PGM1 promoter were presented. Two vertical lines represent two FOXJ2 binding motifs. Red arrows represents transcription start site. (B) ChIP analyses with an anti-p53 antibody were performed in SK-Hep1 cells. Data represent the means ± SD of 3 independent experiments. (C) SK-Hep1 or HepG2 cells were treated with or without 10 ng/mL TGFβ, and immunoblotting analyses were performed in these cells. Underlying data can be found in S1 Data. ChIP, chromatin immunoprecipitation; FBM, FOXJ2-binding motifs; FOXJ2, forkhead box protein J2; HCC, hepatocellular carcinoma; PGM1, phosphoglucomutase 1; TGFβ, transforming growth factor β.
(TIF)

S5 Fig FOXJ2 disrupts the binding of CNBP to PGM1 promoter. Related to Fig 5. Immunoblotting analyses were performed with the indicated antibodies. (A) EV or Flag-FOXJ2 was immunoprecipitated from SK-Hep1 cells stably expressing Vector or Flag-FOXJ2 with anti-Flag antibody. The immunoprecipitated complex was further analyzed by LC-MS for identification of FOXJ2-associated proteins. FOXJ2-associated proteins were listed in a descending order according to the intensity. (B) SK-Hep1 cells were infected with the lentivirus expressing EV or Flag-CNBP. (C) SK-Hep1 and HepG2 cells were harvested for immunoblotting analyses. (D) IHC staining with anti-CNBP antibody was performed in 69 pairs of tumor tissues and corresponding peritumoral tissues from HCC patients. Representative images of tumor tissues and paired peritumoral tissues are shown in top panel. Semiquantitative scoring was performed (bottom panel, paired *t* test, two-tailed, *P* < 0.0001). Underlying data can be found in S1 Data. LC-MS, Liquid Chromatography Mass Spectrometry; FOXJ2, forkhead box protein J2; CNBP, cellular nucleic acid-binding protein; PGM1, phosphoglucomutase 1; HCC, hepatocellular carcinoma; EV, empty vector.
(TIF)

S6 Fig FOXJ2-up-regulated PGM1 expression inhibits glycolysis, cell proliferation, and HCC development. Related to Fig 6. Immunoblotting analyses were performed with the indicated antibodies. (A) SK-Hep1 or Huh7 cells stably expressing shNT or shPGM1 were infected with a lentivirus expressing EV or Flag-FOXJ2. Data are representative of at least 3 independent experiments. (B) SK-Hep1 and HepG2 cells were harvested and subjected to immunoblotting analyses. (C–D) HepG2 cells were infected with the lentivirus expressing EV or Flag-PGM1. Immunoblotting analyses were performed in these cells (panel C). Proliferation was examined in these cells (panel D). Data represent the means ± SD of 3 independent experiments. Underlying data can be found in S1 Data. EV, empty vector; FOXJ2, forkhead box protein J2; HCC, hepatocellular carcinoma; PGM1, phosphoglucomutase 1; shNT, nontargeting shRNA; shPGM1, shRNA against PGM1.
(TIF)

S7 Fig PGM1 and FOXJ2 expression correlates with the prognosis of HCC patients. Related to Fig 7. (A) Univariate analysis calculated by the Cox proportional hazards regression model. Univariate analysis showed that serum AFP, liver cirrhosis, TNM, tumor size, tumor number, vascular invasion, PGM1, FOXJ2, and PGM1/FOXJ2 combination were predictors for OS, whereas serum AFP, liver cirrhosis, TNM, tumor size, tumor number, tumor differentiation, vascular invasion, PGM1, FOXJ2, and PGM1/FOXJ2 combination were predictive for TTR. Multivariate Cox regression analysis indicated that serum AFP, liver cirrhosis, tumor size, tumor number, PGM1/FOXJ2 combination were independent prognostic factors for OS, and liver cirrhosis, tumor size, tumor number, PGM1/FOXJ2 combination were independent prognostic factors for TTR in HCC patients. (B) Correlation analyses between the IHC intensities of PGM1, FOXJ2, or CNBP and HBsAg status (representing HBV infection). Underlying data can be found in S1 Data. AFP, α-fetoprotein; CI, confidential interval; FOXJ2, forkhead box protein J2; HBsAg, hepatitis B surface antigen; HCC, hepatocellular carcinoma; HR, hazard ratio; OS, overall survival; PGM1, phosphoglucomutase 1; TTR, time to recurrence.
(TIF)

S1 Data Numerical data used in all the figures. (XLSX)

## Abbreviations

2-DG: 2-Deoxyglucose
CDG1T: CDG syndrome type 1t
ChIP: chromatin immunoprecipitation
CNBP: cellular nucleic acid-binding protein
DMEM: Dulbecco’s Modified Eagle Medium
EHBH: Eastern Hepatobiliary Surgery Hospital
EMSA: electrophoretic mobility shift assay
EV: empty vector
FBM: FOXJ2-binding motif
FBS: fetal bovine serum
FDA: Food and Drug Administration
FFPE: formalin-fixed paraffin-embedded
FOXJ2: forkhead box protein J2
G-1-P: glucose 1-phosphate
G-6-P: glucose 6-phosphate
HBsAg: hepatitis B surface antigen
HCC: hepatocellular carcinoma
HE: hematoxylin–eosin
HIF-1: hypoxia-inducible factor 1
IHC: immunohistochemistry
IOD: integrated optical density
MT: mutant
Pak1: p21-activated kinase 1
PDX: patient-derived xenograft
PGM1: phosphoglucomutase 1
PHKB: glycogen phosphorylase kinase β-subunit
PYGB: glycogen phosphorylase brain form
PYGL: glycogen phosphorylase
shNT: nontargeting shRNA
shPMG1: shRNA against PGM1
shRNA: short hairpin RNA
SRB: sulforhodamine B
TGF: transforming growth factor
UDP-glucose: uracil-diphosphate glucose
WT: wild-type
