# Supplementary figures and images for: Phosphoglucomutase 1 inhibits hepatocellular carcinoma progression by regulating glucose trafficking
Source: PLoS Biol. 2018 Oct 18;16(10):e2006483. doi: 10.1371/journal.pbio.2006483 (PMC6193743; doi:10.1371/journal.pbio.2006483)

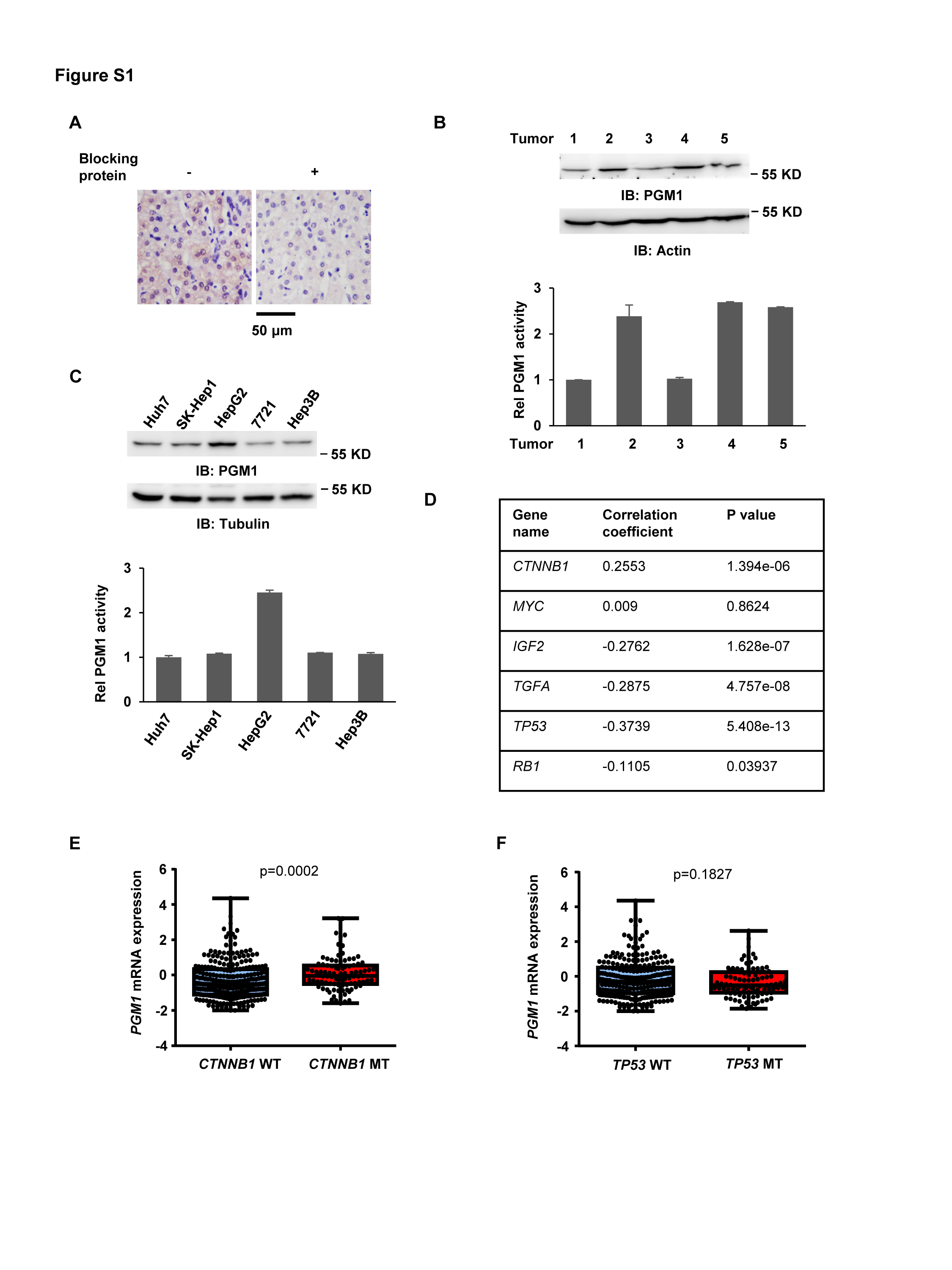

Supplement: S1 Fig — Related to Fig 1. Immunoblotting analyses were performed with the indicated antibodies. (A) IHC analyses of HCC tumor tissues using anti-PGM1 antibody were performed with or without PGM1 blocking protein. (B) PGM1 expression (upper panel) and enzymatic activity (lower panel) were measured in 5 primary HCC tumors. Data represent the means ± SD of 3 independent experiments. (C) PGM1 expression (upper panel) and enzymatic activity (lower panel) were measured in a panel of HCC cell lines. Data represent the means ± SD of 3 independent experiments. (D) Transcriptional correlation analyses (Pearson’s correlation) of PGM1 and some commonly amplified or mutated oncogenes or tumor suppressors in HCC. (E) PGM1 mRNA expression was compared between WT CTNNB1 and MT CTNNB1 samples (Nonsense mutations were excluded). Mann-Whitney test, P = 0.0002. (F) PGM1 mRNA expression was compared between WT TP53 and MT TP53 samples (Nonsense mutations were excluded). Mann-Whitney test, P = 0.1827. Underlying data can be found in S1 Data. CTNNB1, catenin beta-1; HCC, hepatocellular carcinoma; IHC, immunoblotting analyses; MT, mutant; PGM1, phosphoglucomutase 1; TP53, Cellular tumor antigen p53; WT, wild-type. (TIF) [file pbio.2006483.s001.tif]

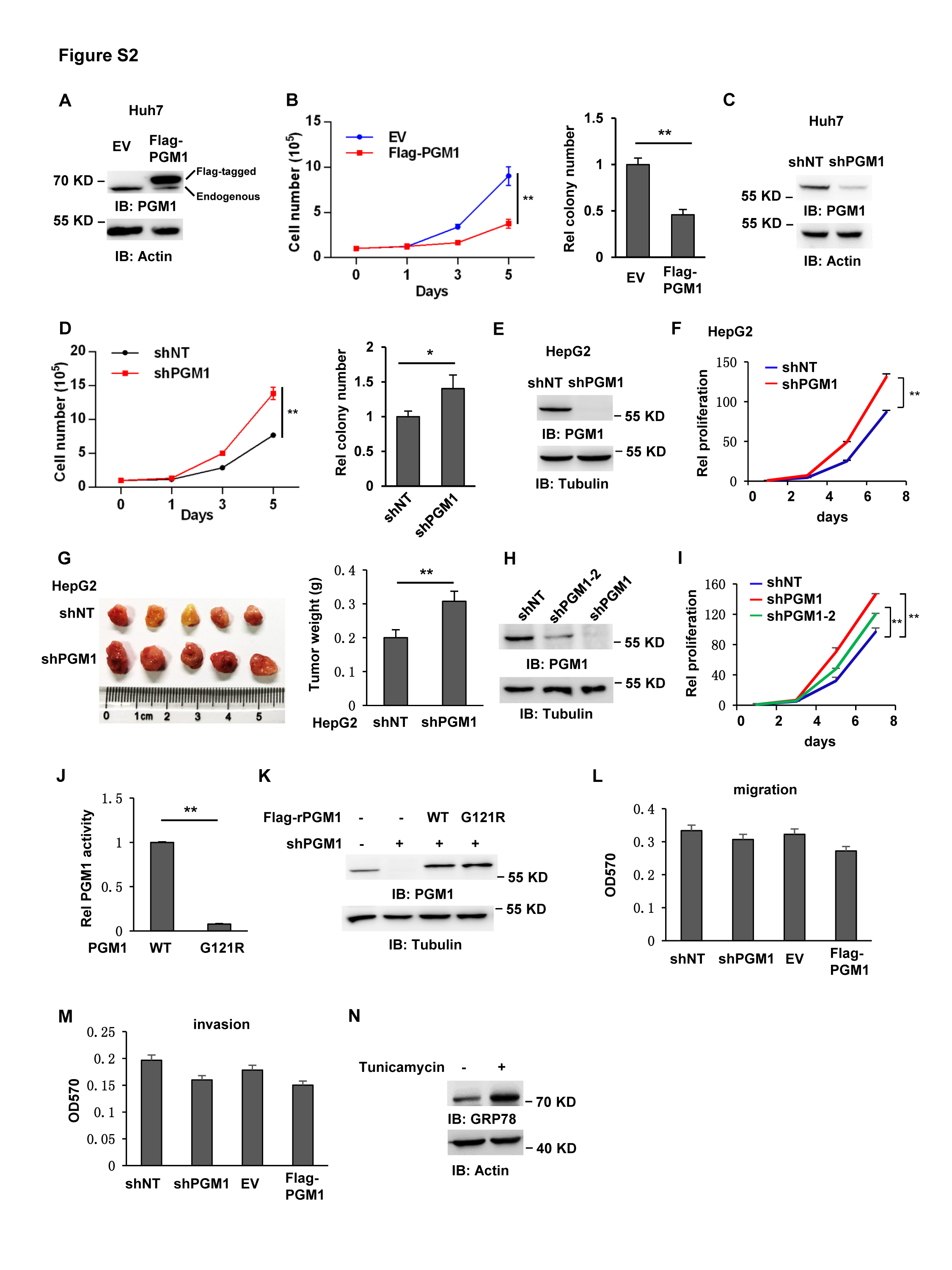

Supplement: S2 Fig — Related to Fig 2. Immunoblotting analyses were performed with the indicated antibodies. (A–B) Huh7 cells were infected with the lentivirus expressing EV or Flag-PGM1. Immunoblotting analyses were performed in these cells (panel A). Proliferation (left panel) and colony formation (right panel) were examined in these cells (panel B). Data represent the means ± SD of 3 independent experiments. (C–D) Huh7 cells were infected with the lentivirus expressing shNT or shPGM1. Immunoblotting analyses were performed in these cells (panel C). Proliferation (left panel) and colony formation (right panel) were examined in these cells (panel D). Data represent the means ± SD of 3 independent experiments. (E–F) HepG2 cells were infected with the lentivirus expressing shNT or shPGM1. Immunoblotting analyses were performed in these cells (panel E). Proliferation (panel F) was examined in these cells using SRB assay. Data represent the means ± SD of 3 independent experiments. (G) Cells in panel E were subcutaneously injected into randomized athymic nude mice (five mice per group). At 30 days after the injection, tumors were dissected for weight measurement. Representative images of dissected tumors are shown in left panel. Quantitative analyses of dissected tumor weights are shown in right panel. Data represent the means ± SD of five mice. (H–I) SK-Hep1 cells were infected with the lentivirus expressing shNT, shPGM1-2 or shPGM1. Immunoblotting analyses (panel H) and proliferation (panel I) were performed in these cells. Data represent the means ± SD of 3 independent experiments. (J) SK-Hep1 cells were infected with the lentivirus expressing Flag-PGM1 WT or G121R. Flag-PGM1 proteins were immunoprecipitated using Flag beads and eluted with Flag peptides to determine PGM1 enzymatic activity. (K) SK-Hep1 cells were depleted of endogenous PGM1 and rescued with Flag-rPGM1 WT or G121R. Immunoblotting analyses were performed in these cells. (L–M) Migration (panel L) and invasion (panel M) of [file pbio.2006483.s002.tif]

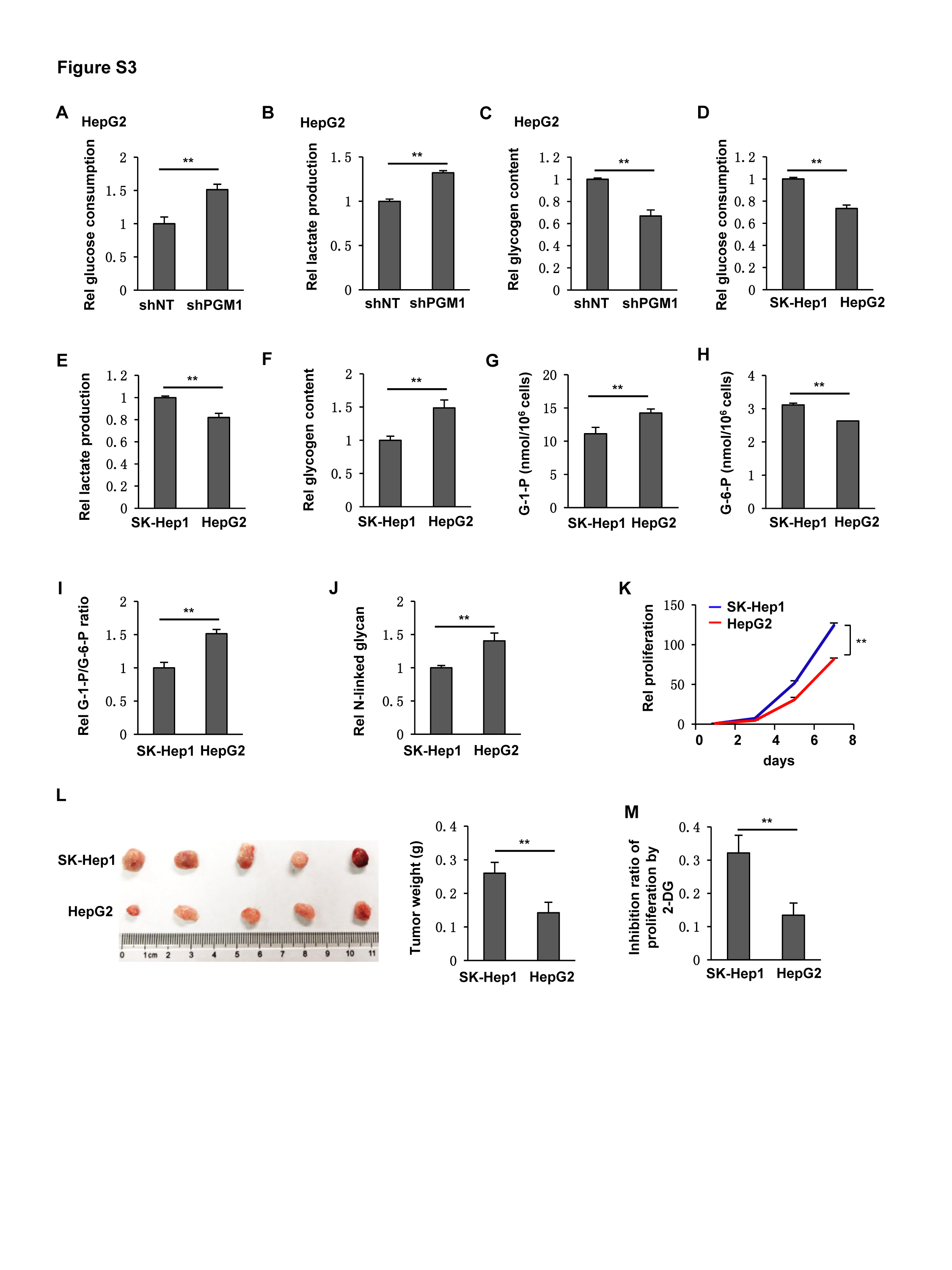

Supplement: S3 Fig — Related to Fig 3. Data represent the means ± SD of 3 independent experiments. (A–C) The culture media of HepG2 cells stably expressing shNT or shPGM1 were collected for analysis of glucose consumption (panel A) and lactate production (panel B). Glycogen content (panel C) of these cells were measured. (D–I) The culture media of SK-Hep1 and HepG2 cells were collected for analysis of glucose consumption (panel D) and lactate production (panel E). Glycogen content (panel F), G-1-P level (panel G), and G-6-P (panel H) of SK-Hep1 and HepG2 cells were measured. G-1-P/G-6-P ratio was calculated (panel I). (J) N-linked glycans of SK-Hep1 and HepG2 cells were measured. (K) Proliferation was examined in SK-Hep1 and HepG2 cells. (L) SK-Hep1 or HepG2 cells were subcutaneously injected into randomized athymic nude mice (five mice per group). At 35 days after the injection, tumors were dissected for weight measurement. Representative images of dissected tumors are shown in left panel. Quantitative analyses of dissected tumor weights are shown in right panel. Data represent the means ± SD of five mice. (M) SK-Hep1 or HepG2 cells were treated with or without 0.5 mM 2-DG, and proliferation of these cells was examined. Underlying data can be found in S1 Data. 2-DG, 2-Deoxyglucose; G-1-P, glucose 1-phosphate; G-6-P, glucose 6-phosphate; PGM1, phosphoglucomutase 1; shNT, nontargeting shRNA; shPGM1, shRNA against PGM1; shRNA, short hairpin RNA. (TIF) [file pbio.2006483.s003.tif]

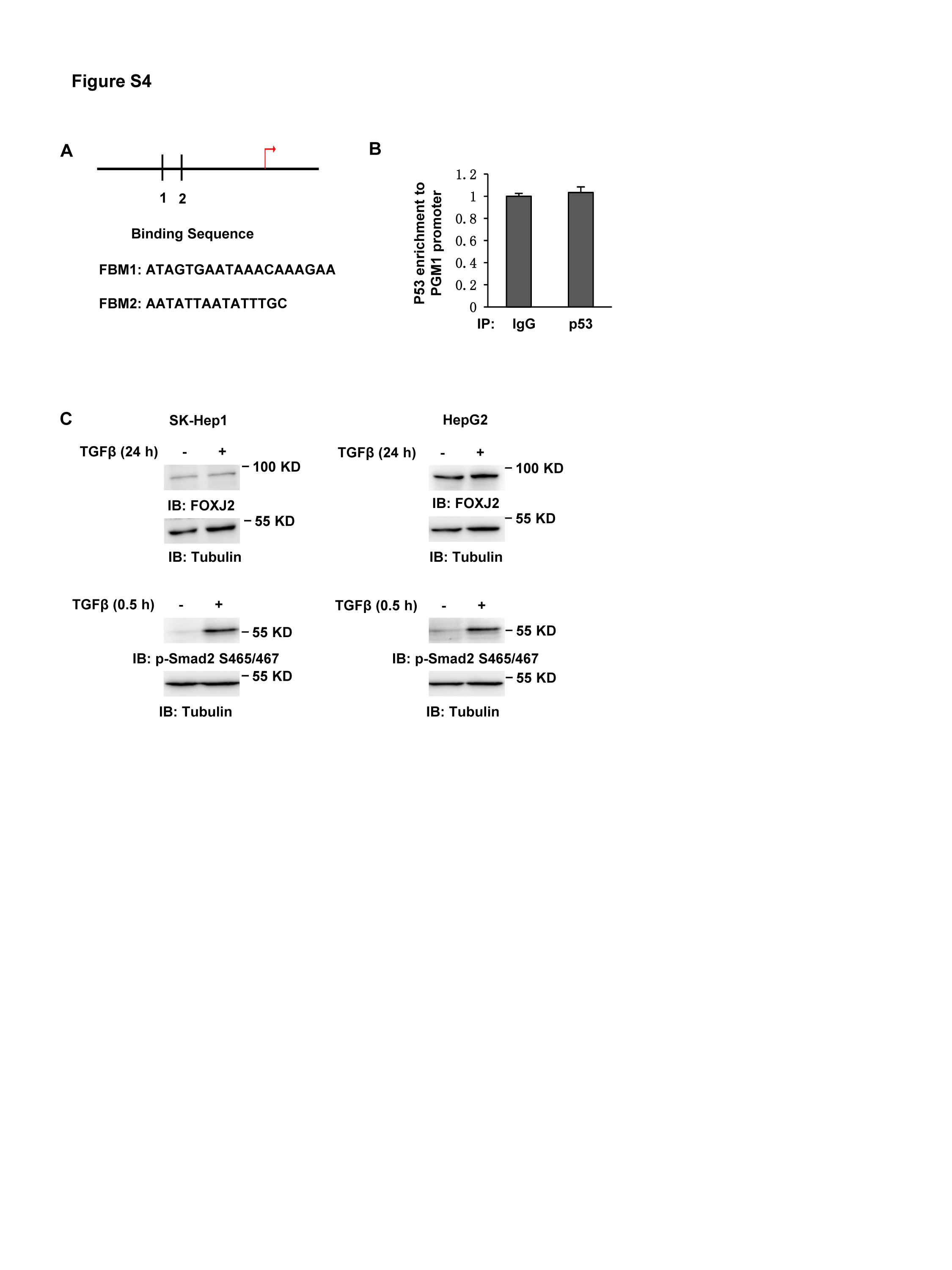

Supplement: S4 Fig — Related to Fig 4. Immunoblotting analyses were performed with the indicated antibodies. (A) Sequences of two FOXJ2 binding motifs (FBM) 1 and 2 in PGM1 promoter were presented. Two vertical lines represent two FOXJ2 binding motifs. Red arrows represents transcription start site. (B) ChIP analyses with an anti-p53 antibody were performed in SK-Hep1 cells. Data represent the means ± SD of 3 independent experiments. (C) SK-Hep1 or HepG2 cells were treated with or without 10 ng/mL TGFβ, and immunoblotting analyses were performed in these cells. Underlying data can be found in S1 Data. ChIP, chromatin immunoprecipitation; FBM, FOXJ2-binding motifs; FOXJ2, forkhead box protein J2; HCC, hepatocellular carcinoma; PGM1, phosphoglucomutase 1; TGFβ, transforming growth factor β. (TIF) [file pbio.2006483.s004.tif]

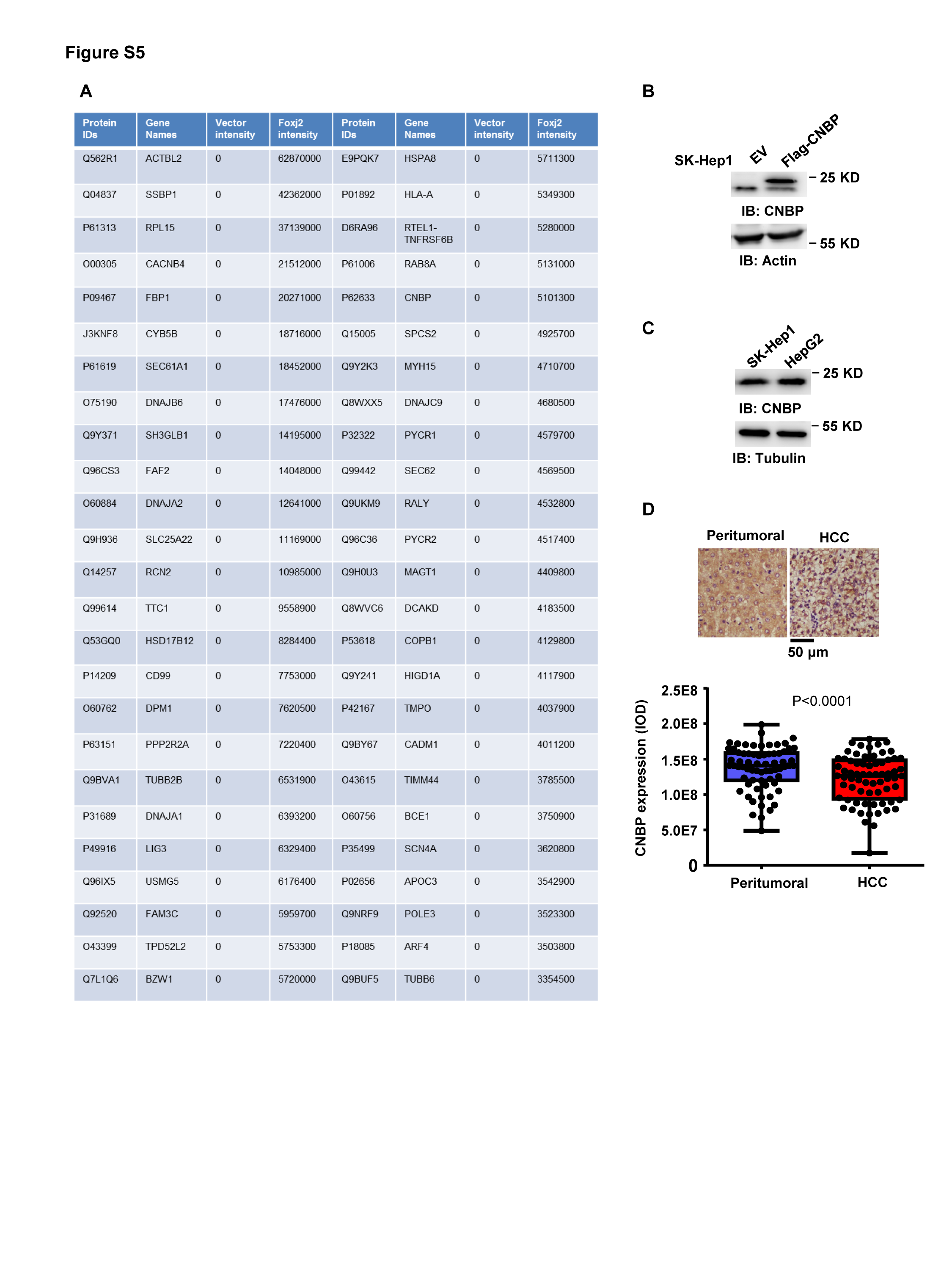

Supplement: S5 Fig — Related to Fig 5. Immunoblotting analyses were performed with the indicated antibodies. (A) EV or Flag-FOXJ2 was immunoprecipitated from SK-Hep1 cells stably expressing Vector or Flag-FOXJ2 with anti-Flag antibody. The immunoprecipitated complex was further analyzed by LC-MS for identification of FOXJ2-associated proteins. FOXJ2-associated proteins were listed in a descending order according to the intensity. (B) SK-Hep1 cells were infected with the lentivirus expressing EV or Flag-CNBP. (C) SK-Hep1 and HepG2 cells were harvested for immunoblotting analyses. (D) IHC staining with anti-CNBP antibody was performed in 69 pairs of tumor tissues and corresponding peritumoral tissues from HCC patients. Representative images of tumor tissues and paired peritumoral tissues are shown in top panel. Semiquantitative scoring was performed (bottom panel, paired t test, two-tailed, P < 0.0001). Underlying data can be found in S1 Data. LC-MS, Liquid Chromatography Mass Spectrometry; FOXJ2, forkhead box protein J2; CNBP, cellular nucleic acid-binding protein; PGM1, phosphoglucomutase 1; HCC, hepatocellular carcinoma; EV, empty vector. (TIF) [file pbio.2006483.s005.tif]

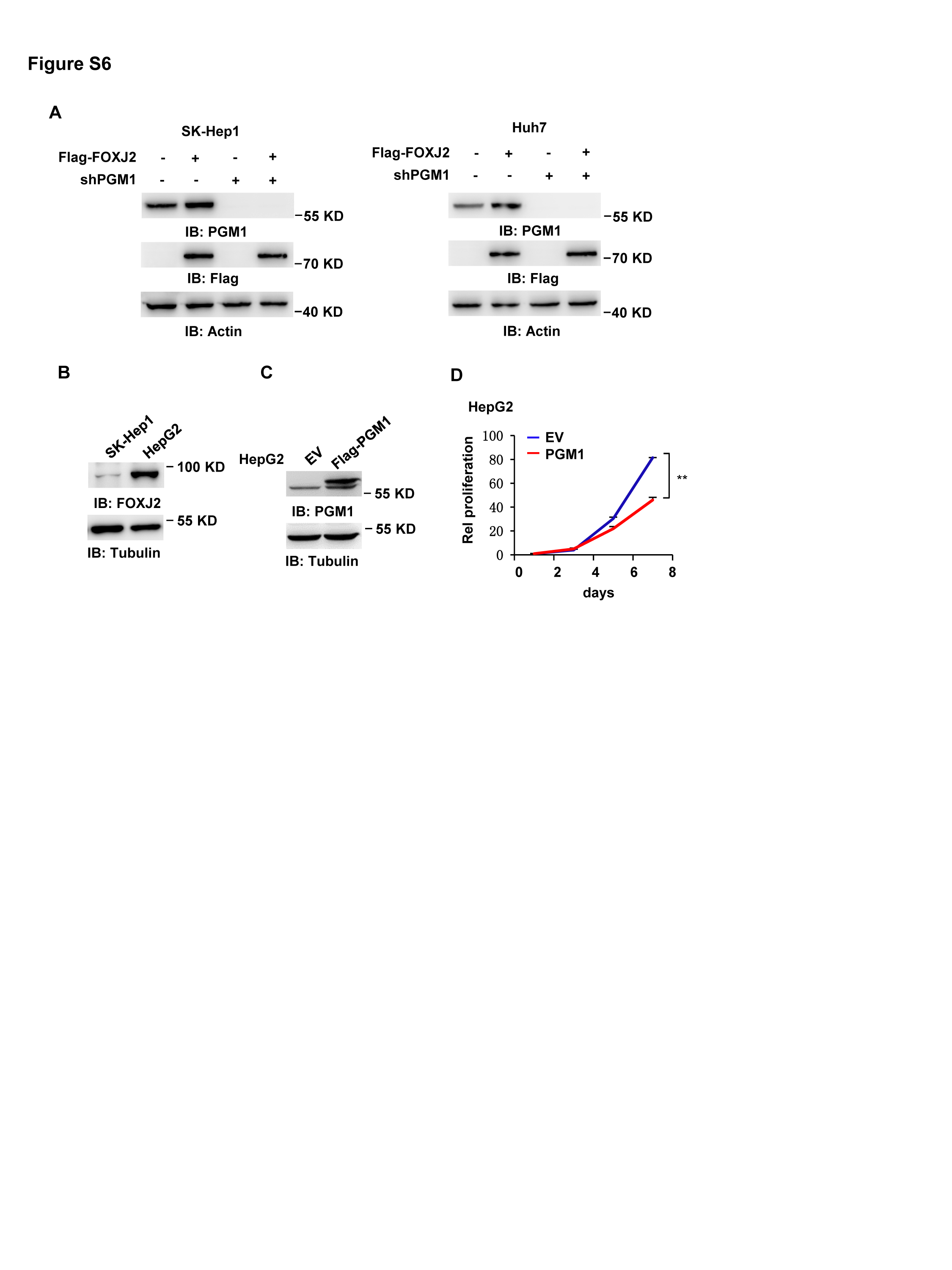

Supplement: S6 Fig — Related to Fig 6. Immunoblotting analyses were performed with the indicated antibodies. (A) SK-Hep1 or Huh7 cells stably expressing shNT or shPGM1 were infected with a lentivirus expressing EV or Flag-FOXJ2. Data are representative of at least 3 independent experiments. (B) SK-Hep1 and HepG2 cells were harvested and subjected to immunoblotting analyses. (C–D) HepG2 cells were infected with the lentivirus expressing EV or Flag-PGM1. Immunoblotting analyses were performed in these cells (panel C). Proliferation was examined in these cells (panel D). Data represent the means ± SD of 3 independent experiments. Underlying data can be found in S1 Data. EV, empty vector; FOXJ2, forkhead box protein J2; HCC, hepatocellular carcinoma; PGM1, phosphoglucomutase 1; shNT, nontargeting shRNA; shPGM1, shRNA against PGM1. (TIF) [file pbio.2006483.s006.tif]

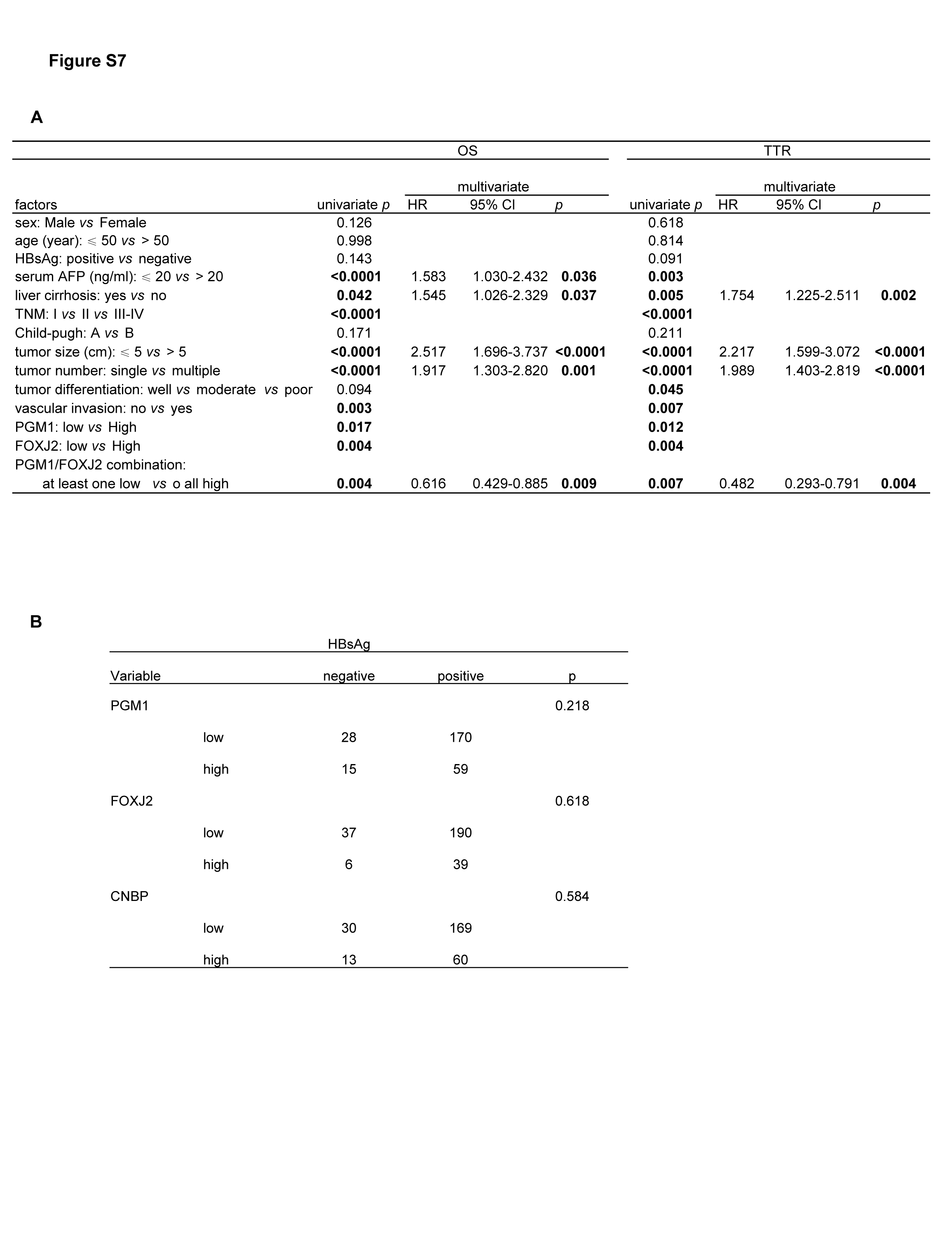

Supplement: S7 Fig — Related to Fig 7. (A) Univariate analysis calculated by the Cox proportional hazards regression model. Univariate analysis showed that serum AFP, liver cirrhosis, TNM, tumor size, tumor number, vascular invasion, PGM1, FOXJ2, and PGM1/FOXJ2 combination were predictors for OS, whereas serum AFP, liver cirrhosis, TNM, tumor size, tumor number, tumor differentiation, vascular invasion, PGM1, FOXJ2, and PGM1/FOXJ2 combination were predictive for TTR. Multivariate Cox regression analysis indicated that serum AFP, liver cirrhosis, tumor size, tumor number, PGM1/FOXJ2 combination were independent prognostic factors for OS, and liver cirrhosis, tumor size, tumor number, PGM1/FOXJ2 combination were independent prognostic factors for TTR in HCC patients. (B) Correlation analyses between the IHC intensities of PGM1, FOXJ2, or CNBP and HBsAg status (representing HBV infection). Underlying data can be found in S1 Data. AFP, α-fetoprotein; CI, confidential interval; FOXJ2, forkhead box protein J2; HBsAg, hepatitis B surface antigen; HCC, hepatocellular carcinoma; HR, hazard ratio; OS, overall survival; PGM1, phosphoglucomutase 1; TTR, time to recurrence. (TIF) [file pbio.2006483.s007.tif]
